# Persistent activation of interlinked type 2 airway epithelial gene networks in sputum-derived cells from aeroallergen-sensitized symptomatic asthmatics

**DOI:** 10.1038/s41598-018-19837-6

**Published:** 2018-01-24

**Authors:** Anya C. Jones, Niamh M. Troy, Elisha White, Elysia M. Hollams, Alexander M. Gout, Kak-Ming Ling, Anthony Kicic, Stephen M. Stick, Peter D. Sly, Patrick G. Holt, Graham L. Hall, Anthony Bosco

**Affiliations:** 1 Telethon Kids Institute, The University of Western Australia, Perth, Australia; 2 School of Paediatrics and Child Health, The University of Western Australia, Perth, Australia; 3 Department of Respiratory Medicine, Princess Margaret Hospital for Children, Perth, Australia; 4 Centre for Cell Therapy and Regenerative Medicine, School of Medicine and Pharmacology, The University of Western Australia, Perth, Australia; 5 Child Health Research Centre, The University of Queensland, Brisbane, Australia; 6 School of Physiotherapy and Exercise Science, Curtin University, Perth, Australia; 7 Centre of Child Health Research, The University of Western Australia, Perth, Australia

## Abstract

Atopic asthma is a persistent disease characterized by intermittent wheeze and progressive loss of lung function. The disease is thought to be driven primarily by chronic aeroallergen-induced type 2-associated inflammation. However, the vast majority of atopics do not develop asthma despite ongoing aeroallergen exposure, suggesting additional mechanisms operate in conjunction with type 2 immunity to drive asthma pathogenesis. We employed RNA-Seq profiling of sputum-derived cells to identify gene networks operative at baseline in house dust mite-sensitized (HDM^S^) subjects with/without wheezing history that are characteristic of the ongoing asthmatic state. The expression of type 2 effectors (IL-5, IL-13) was equivalent in both cohorts of subjects. However, in HDM^S^-wheezers they were associated with upregulation of two coexpression modules comprising multiple type 2- and epithelial-associated genes. The first module was interlinked by the hubs EGFR, ERBB2, CDH1 and IL-13. The second module was associated with CDHR3 and mucociliary clearance genes. Our findings provide new insight into the molecular mechanisms operative at baseline in the airway mucosa in atopic asthmatics undergoing natural aeroallergen exposure, and suggest that susceptibility to asthma amongst these subjects involves complex interactions between type 2- and epithelial-associated gene networks, which are not operative in equivalently sensitized/exposed atopic non-asthmatics.

## Introduction

Asthma is a chronic disease of the conducting airways that is characterized by episodic airways inflammation, airways remodeling, and progressive loss of lung function. It is increasingly recognized as a highly heterogeneous disorder comprising multiple sub-phenotypes^1^. The atopic form of the disease develops in early childhood, and is initiated by sensitization to inhalant allergens exemplified by house dust mite (HDM). One of the important drivers of progression of atopic asthma towards chronicity is thought to be repeated cycles of airways inflammation, in particular severe exacerbations triggered by respiratory infections which involve interactions between host anti-viral and atopy-associated effector mechanisms^2,3^, and the rate of the ensuing decline in lung function is related to the frequency and intensity of these exacerbations^4–6^. There is also evidence to suggest that airway remodeling can proceed independent of these inflammatory processes^7^ but addressing these pathways was beyond the scope of this investigation.

Recent clinical intervention studies, including those demonstrating that treatment with anti-IgE reduces exacerbation frequency, confirms the causal role of type 2 responses in these intermittent events^8–10^, and these findings have provided the impetus for the present study. In particular, the degree to which chronic exposure to type 2-stimulatory perennial aeroallergens contributes to the inflammatory milieu in the airway mucosa of sensitized atopics during the periods between overt exacerbation events, thus potentially influencing long-term persistence of the asthma-associated wheezy phenotype, remains unclear, and this question was the focus of this investigation. Resolving this issue is important in relation to design of future therapeutic strategies for prevention of asthma progression i.e. is it sufficient to target severe exacerbation events alone, or is it potentially necessary to also dampen ongoing aeroallergen-driven type 2 reactivity at baseline in sensitized/perennially exposed subjects?

We have addressed this issue in a study population consisting of 22 yr olds from an unselected birth cohort resident in Perth, Western Australia^11^. We have previously shown that the dominant asthma-associated aeroallergen in this region is HDM^12^ which is present in local households at high levels throughout the year^13^, and accordingly the study focused primarily on atopics who were sensitized and chronically exposed to HDM. Our approach was based on the recent demonstration that induced sputum, which contains a sample of cell populations present on the airway surface, can potentially be used for gene expression profiling of wheeze-associated inflammatory responses in asthmatics^14,15^.

We hypothesized that the presence versus absence of current wheezing history amongst HDM^S^ subjects will be reflected by variations in gene network patterns amongst cell populations accessible at the airway epithelial surface. To test this hypothesis, we have employed RNA-Seq in conjunction with coexpression network analysis to profile asthma-associated gene networks in sputum samples collected at (symptom-free) baseline from study groups matched for age, HDM sensitization status and environmental exposure, but dichotomous with respect to wheezing symptom expression. Our findings suggest that upregulation of type 2 signature genes exemplified by the effector cytokines IL-5 and IL-13 is a common feature across the whole HDM^S^/exposed population at baseline, but in the subgroup with history of current wheeze the type 2 signature is more complex, and is uniquely networked with a series of concomitantly upregulated epithelial cell associated pathways.

## Results

### Demographics of the study population

The study was based on case/control comparisons of HDM^S^ or nonatopic subjects with or without a history of wheeze (Table 1). A total of 68 high quality (cell viability >48%; squamous cell contamination <32%; RNA integrity number > 6) sputum samples were available for transcriptome analysis (Supplementary Fig. S1). The characteristics of the 4 study groups are illustrated in Table 1. There was no difference in age, gender, height, or weight between the four groups. Except for cat, the prevalence of positive skin prick tests to common allergens was not different between HDM^S^ wheezers and HDM^S^ nonwheezers. Asthma medication use was significantly higher in the HDM^S^ wheezers for inhaled short-acting beta-agonists (p < 0.001) and combination therapy (p = 0.004) (Supplementary Table S1).

**Table 1:** Characteristics of the study population.

	Nonatopic	Nonatopic	HDM^S^	HDM^S^	P
	controls	wheezers	nonwheezers	wheezers	
**Number of participants**	**21**	**7**	**24**	**16**	
Male (%)	42.9	42.9	54.2	56.3	0.804
Wheeze in past 12 months [A] (%)	0.0	100.0	0.0	100.0	
Doctor diagnosis of asthma ever [B] (%)	9.5 a	57.1 b,c	41.7 a,c	93.8 b	<0.001
Asthma medication use in past 12 months [C] (%)	4.8 a	14.3 a	8.3 a	75.0 b	<0.001
Current medicated asthma [Postive for A,B&C] (%)	0.0	14.3	0.0	75.0	0.007^
Current asthma [Positive for A&B] (%)	0.0	57.1	0.0	93.8	0.033^
Airways hyperresponsiveness (%)	0.0	0.0	0.0	43.7	<0.001
Parental history of asthma at recruitment (%)	10.0*	28.6	21.7*	37.5	0.263
Current rhinoconjunctivitis (%)	0.0 a	42.9 b	0.0 a	75.0 b	<0.001
Current smokers (%)	23.8	14.3	33.3	25.0	0.740
Height (m)	1.76 [1.65–1.79]	1.70 [1.59–1.78]	1.76 [1.66–1.82]	1.73 [1.60–1.84]	0.415
Weight (kg)	67.6 [61.5–82.9]	76.9 [56.0–83.0]	75.1 [66.4–89.7]	70.3 [60.9–102.4]	0.561
Hip to waist ratio	0.83 [0.79–0.88]	0.83 [0.78–0.85]	0.85 [0.81–0.90]	0.87 [0.77–0.88]	0.754
Age at assessment and sputum collection (years)	22.0 [21.8–22.2]	21.9 [21.6–21.9]	22.2 [21.6–22.5]	22.0 [21.6–22.5]	0.660
Prevalence of positive skin prick tests (%)					
Any positive	0.0	0.0	100.0	100.0	1.000^^
HDM	0.0	0.0	100.0	100.0	1.000^^
Egg white	0.0	0.0	4.2	12.5	0.327^^
Mold	0.0	0.0	16.7	25.0	0.519^^
Grass mix	0.0	0.0	45.8	50.0	0.796^^
Cow’s milk	0.0	0.0	0.0	6.3	0.215^^
Grass pollen	0.0	0.0	50.0	50.0	1.000^^
Dog	0.0	0.0	20.8	31.3	0.456^^
Cockroach	0.0	0.0	29.2	50.0	0.182^^
Fungus	0.0	0.0	12.5	18.8	0.588^^
Cat	0.0	0.0	33.3	75.0	0.0106^^
HDM SPT wheal diameter (mm):					
*Sum of D*. *pteronyssinus and D*. *farinae*	0.0 [0.0–0.0]	0.0 [0.0–0.0]	10.3 [6.0–17.3]	15.9 [10.0–20.2]	0.062^^
Participants with IgE data (n):	19	7	23	14	
HDM-IgE ≥ 0.35 kU/L (%)	5.3 a	14.3 a,b	69.6 b,c	100 c	<0.001
Baseline lung function measures from all participants:					
FEV_1_	4.0 [3.4–4.5]	3.6 [3.2–4.9]	4.2 [3.4–4.6]	3.5 [2.9–5.0]	0.809
FEV_1_% of predicted	100.1 [88.6–102.9]	104.3 [97.7–105.1]	96.1 [89.1–106.5]	93.2 [85.5–102.6]	0.445
FEV_1_ (z score)	0.0 [−1–0.3]	0.4 [−0.2–0.4]	−0.3 [−0.9–0.6]	−0.6 [−1.2–0.2]	0.445
FVC	4.7 [3.7–5.7]	4.2 [3.8–5.8]	5.1 [4.3–5.9]	4.6 [3.7–6.4]	0.767
FVC % of predicted	96.6 [94.5–106.2]	104.0 [96.3–111.6]	100.5 [95.9–109.6]	100.9 [89.0–105.9]	0.596
FVC (z score)	−0.3 [−0.5–0.5]	0.3 [−0.3–1.0]	0.0 [−0.4–0.8]	0.1 [−0.9–0.5]	0.627
FEV_1_/FVC	0.85 [0.80 −.0.89]	0.88 [0.79–0.91]	0.84 [0.77–0.87]	0.79 [0.76–0.84]	0.136
FEV_1_/FVC % of predicted	0.87 [0.85–0.88]	0.87 [0.85–0.88]	0.86 [0.85–0.87]	0.86 [0.85–0.88]	0.608
Forced exhaled nitric oxide, ppb	25.5 [19.5–30.0] a	24.0 [17.9–39.1] a	31.5 [22.2–58.2] a	47.2 [35.1–88.7] b	0.004

Median [interquartile range] is displayed for all continuous measures. P value is derived from analyses comparing the four groups: prevalence values were compared by Chi square analysis; continuous measures were compared by Kruskal Wallis analysis. Where significant differences were observed between the four group groups (P < 0.05 in table), each letter denotes sputum groups that do not differ significantly at the 0.05 level after adjusting for multiple pair-wise comparisons (e.g. a vs a = not significantly different, a vs b = significantly different). *Data was missing for 1 participant in each group. ^Wheezing groups only were compared by Mann Whitney U test. ^^Atopic groups only were compared by Chi square (IgE binary variables) or Mann Whitney U test (SPT wheal diameter). Skin prick test were classified positive if wheal diameter was ≥3 mm. Baseline spirometry data are reported in absolute terms, in addition to z score and predicted terms that were calculated using the 2012 Global Lung Function Initiative equations^72^.

### Cellular composition of the sputum

The cellular composition of sputum from these subjects was dominated by macrophages and neutrophils, which constituted 88–94% of the overall population, and the proportion of these cell types (and overall total yields) did not differ between the groups (Supplementary Table S2). Squamous cells and lymphocytes comprised on average 4.5% and 1.8% respectively and did not differ between groups. Small numbers of eosinophils were detectable only in the atopic groups and were highest in the wheezers (Supplementary Table S2).

### Differential gene expression in HDM^S^ nonwheezers

**Figure 1:**
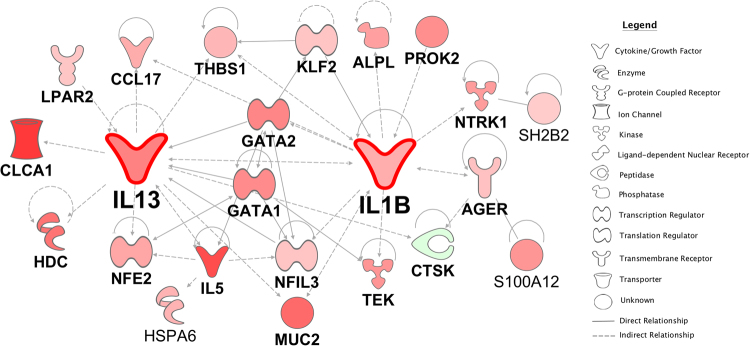
Differential gene network comparing HDM^S^ nonwheezers versus nonatopic controls. Differentially expressed genes were identified with an edgeR analysis. Gene expression patterns in sputum were compared between HDM^S^ nonwheezers and nonatopic controls. The wiring diagram of the underlying gene network was reconstructed employing prior knowledge from the literature (Ingenuity Knowledge Base). Genes highlighted in red denote upregulation, whilst green indicates downregulation in HDM^S^ nonwheezers.

Gene expression patterns in sputum were firstly compared between HDM^S^ nonwheezers and nonatopic controls. The data showed that 80 genes were upregulated (including the type 2 signature genes IL-5 [4.15 logfold] and IL-13 [2.92 logfold]) and 11 genes were downregulated (FDR < 0.05, Supplementary Table S3). To obtain detailed information on the regulatory interactions between these genes, we utilized experimentally supported findings from published studies (prior knowledge) to reconstruct the underlying network^16^. It is noteworthy that this analysis includes relationships such as binding interactions between proteins, and regulatory interactions between transcription factors and their target genes. This analysis showed that the genes were mainly involved in IL-1B and IL-5/IL-13 signaling (Fig. 1).

### Differential gene expression in HDM^S^ wheezers

**Figure 2:**
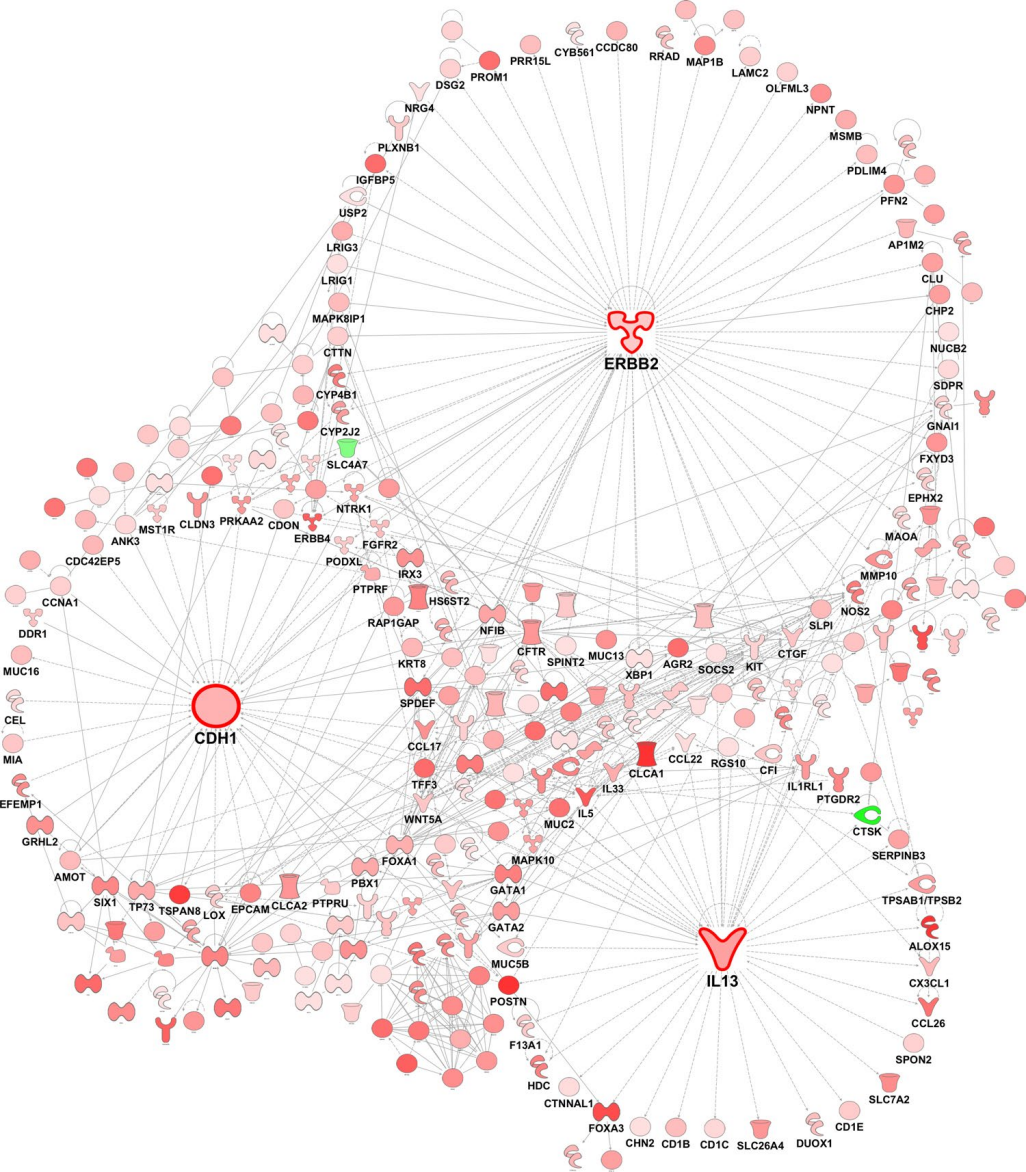
Differential gene network comparing HDM^S^ wheezers versus nonatopic controls. Differentially expressed genes were identified with an edgeR analysis. Gene expression patterns in sputum were compared between HDM^S^ wheezers and nonatopic controls. The wiring diagram of the underlying gene network was reconstructed using prior knowledge from the literature (Ingenuity Knowledge Base). Genes highlighted in red denote upregulation, whilst molecules in green indicate downregulation in HDM^S^ wheezers.

Next we compared gene expression patterns between HDM^S^ wheezers and nonatopic controls. The data showed that 842 genes were upregulated (again including IL-5 [5.29 logfold], IL-13 [3.03 logfold] and IL-33 [2.59 logfold]) and 11 genes were downregulated in the wheezers (FDR < 0.05, Supplementary Table S4). As illustrated in Fig. 2, reconstruction of the prior knowledge network revealed that these genes revolved around a few hubs - erb-b2 receptor tyrosine kinase 2 (ERBB2/HER2), which was involved in 59 interactions (also known as ‘edges’ in graph theory^17^), IL-13 (44 edges), and E-Cadherin/CDH1 (37 edges).

**Figure 3:**
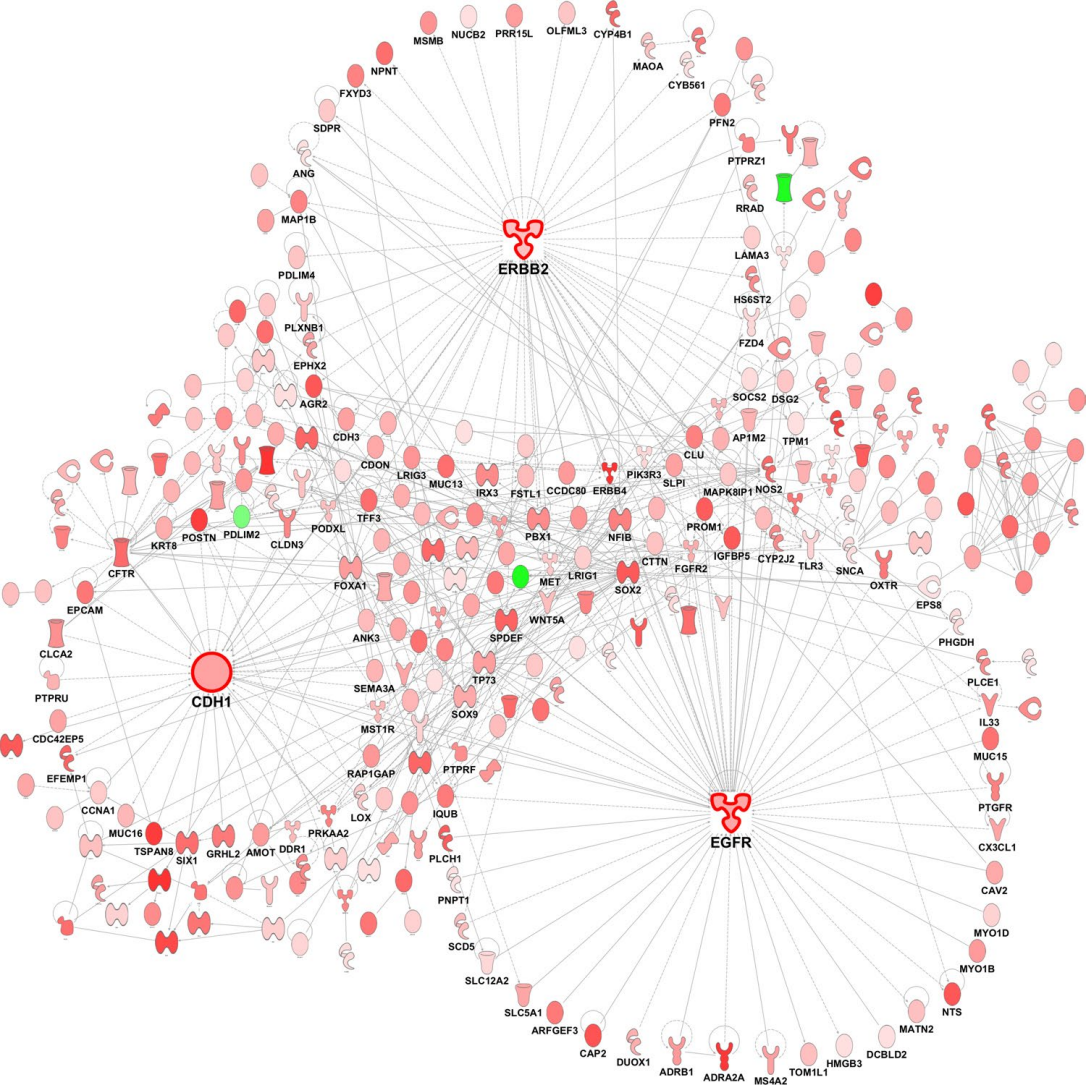
Differential gene network comparing HDM^S^ wheezers versus HDM^S^ nonwheezers. Differentially expressed genes were identified with an edgeR analysis. Gene expression patterns in sputum were compared between HDM^S^ wheezers versus HDM^S^ nonwheezers. The wiring diagram of the underlying gene network was reconstructed employing prior knowledge from the literature (Ingenuity Knowledge Base). Genes highlighted in red denote upregulation, whilst molecules in green indicate downregulation in HDM^S^ wheezers.

Further, we compared gene expression patterns between HDM^S^ wheezers versus HDM^S^ nonwheezers. The data showed that 859 genes were upregulated and 8 genes were downregulated (FDR < 0.05, Supplementary Table S5). The prior knowledge network constructed from these genes (Fig. 3) unveiled epidermal growth factor receptor (EGFR, 60 edges), ERBB2 (56 edges) and CDH1 (38 edges) as hub genes. It is noteworthy that IL-13 did not feature here since it was not differentially expressed after adjustment for multiple testing (p-value = 0.028, FDR = 0.27). In contrast, IL-33 was upregulated in this comparison (2.74 logfold; Supplementary Table S5).

Finally, we compared gene expression in nonatopic wheezers with nonatopic controls, and a single gene - LIM domain binding 3 (LDB3), was upregulated in the subjects with wheeze (FDR = 2.6 × 10^−5^). Given the lack of differentially expressed genes and the small sample size these data were not considered further.

### Network analysis identifies asthma-associated modules operating at baseline in HDM^S^ wheezers

It has been reported that hub genes in biological interaction networks often exhibit limited expression changes in experimental asthma models^18^, thus a potential caveat of the above analyses, which focused on differentially expressed genes, is that some hubs may have escaped detection. These hubs can be captured using coexpression network analysis. Moreover, network analysis additionally provides a deeper understanding of the molecular context in which differentially expressed genes operate. Hence, we constructed a genome-wide coexpression network, utilizing the data from both HDM^S^ groups (n = 40). The resulting network comprised 14,833 genes organized into 23 coexpression modules. To identify disease-associated modules, we plotted the −log10 p-values derived from the above differential expression analyses on a module-by-module basis. The data showed that the modules were not different between HDM^S^ nonwheezers and nonatopic controls (Supplementary Fig. S4A). In contrast, three modules (designated A, P, and Q) were upregulated in HDM^S^ wheezers versus the other two groups (nonatopic controls, HDM^S^ nonwheezers, Supplementary Fig. S4B,C).

### Module reconstruction reveals hub genes in HDM^S^ wheezers

**Figure 4:**
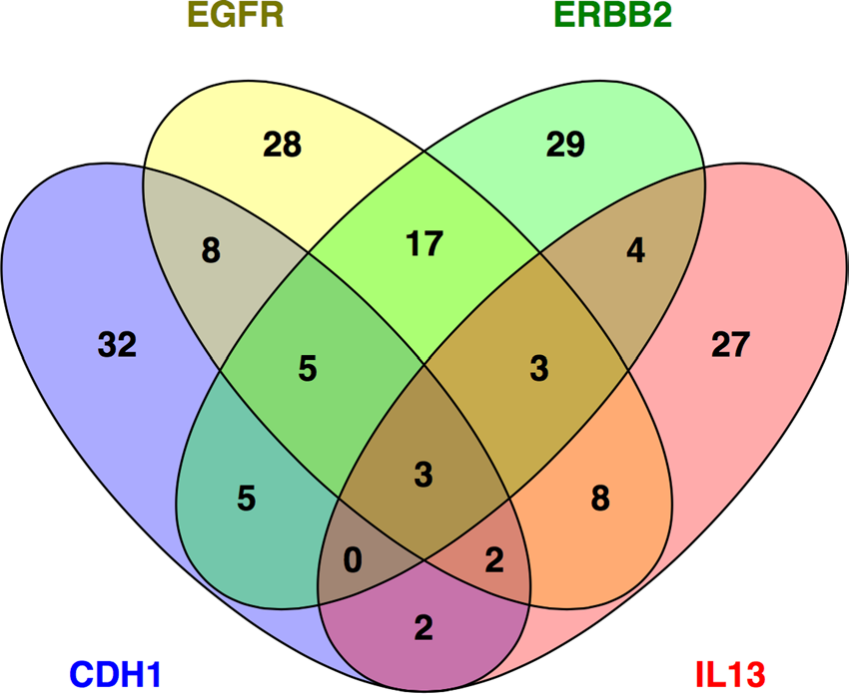
Common and unique genes networked with the 4 hub genes: EGFR, ERBB2, CDH1 and IL-13. Venn diagram illustrating common and unique genes that are networked with each hub gene in merged modules “P” and “Q”.

**Figure 5:**
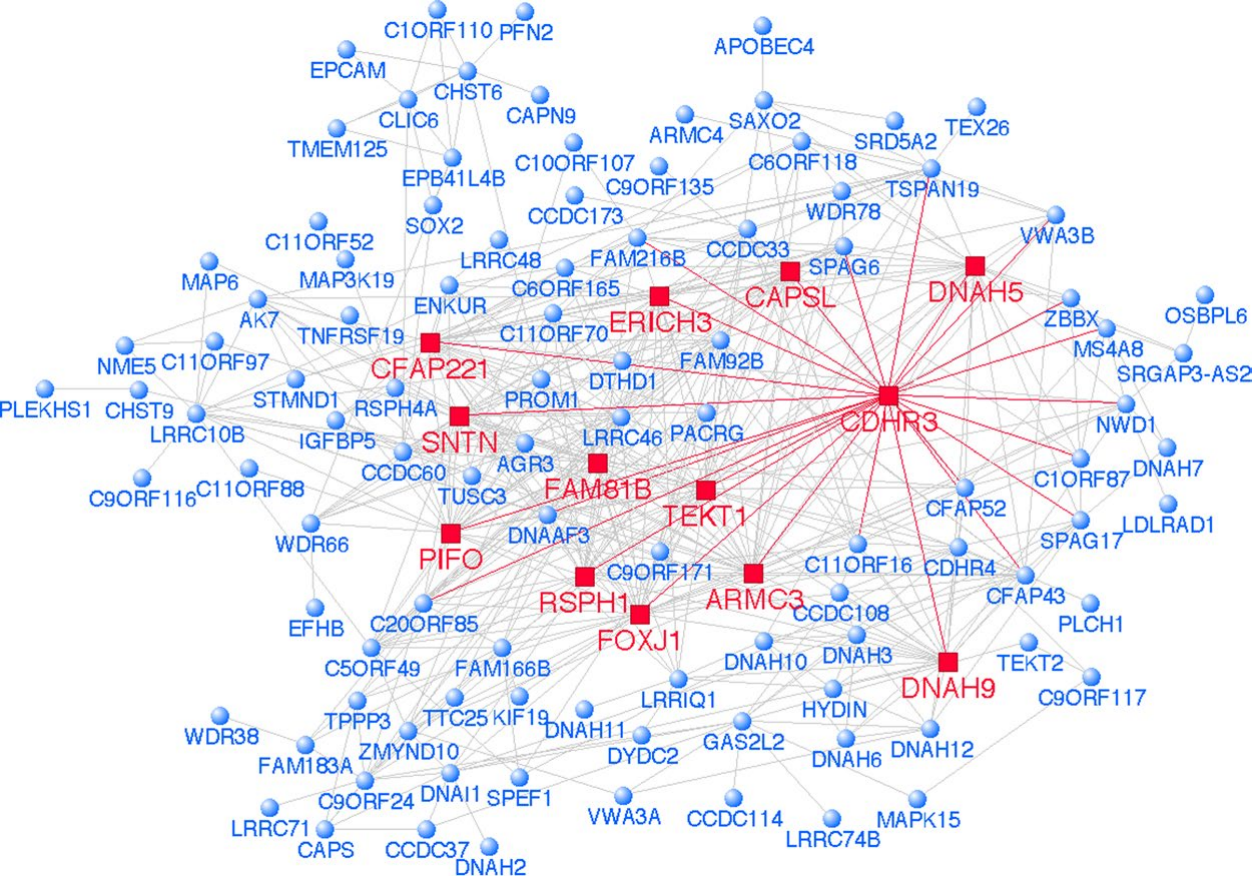
Differential gene network comparing HDM^S^ wheezers versus nonatopic controls/HDM^S^ nonwheezers. Gene co-expression network analysis (WGCNA) was employed to construct the gene networks and unbiased correlation patterns were utilized to reconstruct the underlying wiring diagram of the mucociliary clearance module “A”. CDHR3 is identified as a hub and dominant hubs are highlighted in red.

Module “P” contained 319 genes, and based on prior knowledge the dominant hubs in the network were EGFR (35 edges) and CDH1 (31 edges, Supplementary Fig. S5). Module “Q” contained 440 genes, and the hubs in the reconstructed prior knowledge network were ERBB2 (35 edges) and IL-13 (27 edges, Supplementary Fig. S6). Principal component analysis showed that these two modules (P, Q) were highly correlated (Pearson correlation: 0.897, p-value = 4.441 × 10^−15^) (Supplementary Fig. S7), suggesting they are subunits of a larger parent module. Therefore, we merged them into a single network. In the merged network the dominant hubs were EGFR (73 edges), ERBB2 (65 edges), CDH1 (56 edges) and IL-13 (48 edges, Supplementary Fig. S8). Notably, these hubs connect to both common and unique pathways (Fig. 4). The biological function of the genes that interact with the hubs was interrogated using Gene Ontology terms (Supplementary Table S6) and Pubmed searches (Supplementary Table S7). Module “A” contained 506 genes. It was not possible to reconstruct this module using prior knowledge, because no interactions were found for the vast majority of genes. Therefore we used unbiased correlation patterns to reconstruct the network^19^. This analysis showed that the highest-ranking coexpression hubs were TEKT1, FOXJ1, ARMC3, PIFO, DNAH5, RSPH1, FAM81B, SNTN, ERICH3, DNAH9, and CAPSL (Fig. 5). Of note, CDHR3, which is a known susceptibility gene for asthma exacerbations and a receptor for rhinovirus species C^20,21^, was a highly ranked coexpression hub (rank 19 out of 506 genes) within module “A”.

### Network module enrichment analysis reveals enrichment for airway epithelial cells

The molecular signatures database^22^ was employed to interrogate the modules for enrichment of gene ontology terms and immunological signatures in the data (Supplementary Table S8). This analysis showed that the CDHR3-associated module (module A) was enriched with genes expressed in ciliated epithelial cells, whereas the other modules contained signatures related to mesenchymal and epithelial cells (cell junction, tissue development, epithelial development). We also employed the Human Gene Atlas to interrogate the data, and module P was most strongly associated with bronchial epithelial cells (p-value = 8 × 10^−9^), and module Q was associated with trachea (p-value = 0.006). Signatures related to T cells, mast cells, and neutrophils were also detected, but these were not highly ranked (data not shown).

### Adjustment of differential expression for potential confounders

Next we determined if the associations between the modules and wheeze amongst HDM^S^ subjects were potentially confounded by variations in cellular composition, smoking, or the use of medications. We counted the proportion of epithelial cells in the two HDM^S^ groups, and the mean proportion was 1.2% (±1.6 SD), and this was not different between wheezers and nonwheezers (p-value = 0.7). We repeated the analyses adjusting for variations in the proportion of squamous cells, and the data were unchanged (Supplementary Fig. S9B). We adjusted the analysis for proportions of eosinophils, and this reduced the number of differentially expressed genes and associated modules (Supplementary Fig. S9C). We adjusted the analysis for smoking, and the data were unchanged (Supplementary Fig. S9D). Finally, to investigate the impact of medications on the data, we employed Upstream Regulator Analysis^23^ to systematically search for steroid-related activation signatures, and no significant signatures were identified (data not shown).

### Replication of the CDHR3-associated mucociliary module “A” in two independent asthmatic datasets

We then wanted to determine if the modules could be replicated in independent data sets. We downloaded two data sets from the gene expression omnibus repository. The first data set was from asthmatic bronchial epithelial brushings (GSE76226), and the second data set was from asthmatic sputum samples (GSE41863). Coexpression analysis of both data sets demonstrated that firstly the CDHR3-associated module (module A) was reproducible (Supplementary Fig. S10), and secondly that CDHR3 was a highly ranked coexpression hub (rank 12 out of 1750 genes, GSE76226; rank 12 out of 271 genes, GSE41863) in both independent data sets. Further, we found that the highest-ranking genes in our dataset that were detected in the independent data sets were amongst the top 15% of coexpression genes in the validation datasets (data not shown). It was not possible to validate the other modules, because the bulk of the microarray probe sets did not pass our stringent filtering criteria (see supplementary methods).

### CDHR3 and EGFR expression is higher in atopic asthmatic bronchial epithelial cells versus nonatopic controls

**Figure 6:**
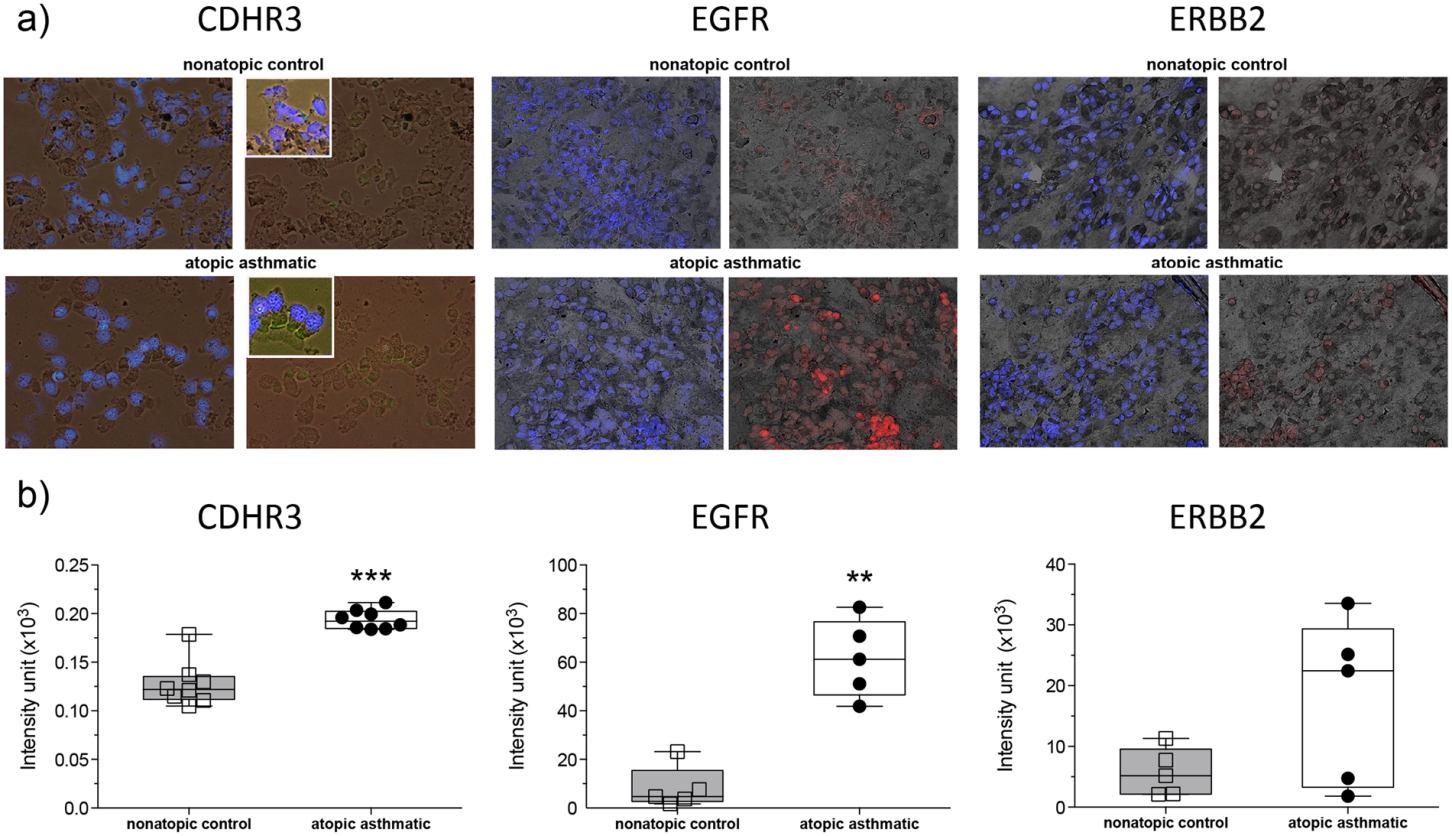
Increased expression of CDHR3 and EGFR in bronchial epithelial cells from HDM sensitized asthmatics versus nonatopic controls. The expression of the hub genes CDHR3, EGFR and ERBB2 was validated at the protein-level in an independent cohort. Bronchial epithelial cells were obtained from HDM sentitized atopics with asthma and nonatopic controls. (**a**) Bronchial epithelial cells were immunofluorescently stained for CDHR3 expression^23^, EGFR expression^71^, ERBB2 expression^71^ and nuclei with DAPI (blue). Staining images were then overlaid over bright field images taken of the same field of view. Note: mag 200×; inset 400×. (**b**) Quantification of the images demonstrated that the expression of CDHR3 and EGFR was more intense in the atopics with asthma. The expression of ERBB2 was not different between the groups. Mann-Whitney U Test was utilized to test for statistical significance. ***p-value < 0.001, **p-value < 0.01.

We finally wanted to validate the expression of selected hubs at the protein-level in an independent cohort of subjects. CDHR3 was selected for these studies because it is a candidate susceptibility gene for asthma exacerbations, but its normal endogenous cellular function is unknown. We also selected EGFR and ERBB2 for validation because these were the dominant hubs in the other modules. These studies focused on bronchial epithelial cells, based on bioinformatics analyses of molecular signatures in the data (Supplementary Table S8). The staining was carried out on samples from HDM sensitized children with asthma and from nonatopic controls. The demographics of the paediatric cohort are presented in Supplementary Table S11. The data for CDHR3 showed there was positive staining localized to the apical surface of columnar epithelial cells in both cohorts (Fig. 6a, Supplementary Fig. S11), and this was more intense and defined in airway epithelial cells derived from the asthmatic children (Fig. 6b). EGFR expression was also increased in the atopic asthmatics (Fig. 6a,b, Supplementary Fig. S12). In contrast, mean expression of ERBB2 (in red) was 3-fold higher in the atopic asthmatics; however, this was not statistically significant because it was only elevated in a subset of the subjects (Fig. 6a,b, Supplementary Fig. S13).

## Discussion

Asthma is acknowledged to be a heterogeneous disease with multiple different phenotypes^1^. Local inflammation and tissue remodeling in the airways are common features of ongoing disease^7^, and there is evidence suggesting that these processes can act independently and/or in concert to drive asthma pathogenesis^7^. The specific focus of this study is asthma in young adult atopics who are highly sensitized to HDM allergen. In this regard, an increasing body of epidemiological and experimental evidence (reviewed^2,3,24^), now supported by a range of intervention studies^4–6^, argues for a causal role for type 2-associated inflammatory mechanisms in the aetiology and pathogenesis of atopic asthma. However, the precise molecular details of the underlying causal pathways remain incompletely understood. In particular, the relative contributions of airways inflammation resulting from acute severe exacerbation events versus chronic exposure to relevant aeroallergens to time-related lung function decline in asthmatics, remains unknown. Moreover, while it is undisputed that sensitization to perennial aeroallergens is an important asthma risk factor, community wide studies clearly demonstrate that only a minority of sensitized subjects including of those highly sensitized to HDM^25^ ever develop persistent wheeze. This suggests that additional cofactor(s) may be required to unmask the full pathogenic potential of aeroallergen-specific sensitization. A likely candidate in this regard is the airway epithelium, which may function as both a target for type 2-associated inflammation and/or as an active participant via production of a range of immunomodulatory molecules that can regulate the local functioning of Th2 cells and group 2 innate lymphoid cells^8–10,26,27^.

Our current study design represents an unbiased approach towards testing this possibility. In the core experiments we have sampled induced sputum cell populations from equivalently sensitized adult atopics undergoing natural aeroallergen exposure, and employed gene expression profiling and ensuing bioinformatics analyses to compare and contrast gene network patterns after stratification on the basis of wheezing phenotypes. Our findings provide novel insight into the nature of the molecular processes ongoing on the airway mucosal surface at the time of sampling.

Our initial analyses showed that a type 2 gene expression program was upregulated in baseline sputum samples from HDM sensitized atopics, regardless of whether these subjects have current history of wheeze. This is consistent with earlier immunohistochemical findings indicating that mast cell degranulation in the airway mucosa is present in both atopic asthmatics and atopic nonasthmatics, albeit to differing degrees^28^. Of note, although asthma medication use was significantly higher in the HDM^S^ wheezers, steroid-related signatures were absent in the transcriptome, likely reflecting the participants abstaining from using their medication 72 hours prior to respiratory assessment and sputum collection, as requested by the study protocol. In the present study, the key type 2 effectors IL-5 and IL-13 were upregulated to comparable degrees in both groups, however in HDM^S^ nonwheezers the overall type 2 program was restricted to a few IL-5/IL-13-associated genes. In contrast, hundreds of genes were upregulated in HDM^S^ wheezers, and network analysis suggested that these genes function in the context of two discrete coexpression modules. Reconstruction of the first module unveiled the hub genes EGFR, ERBB2, CDH1, and IL-13, which dominated the network structure. The second coexpression module comprised genes that control mucociliary clearance, and CDHR3 was identified as a coexpression hub within this module. Overall, our findings suggest that the molecular mechanisms that determine susceptibility to asthma-associated wheeze amongst HDM sensitized atopics involve complex interactions between type 2 and epithelial gene networks. Whilst interactions between type 2 and epithelial cells have previously been implicated in the pathogenesis of atopic asthma^26,27^, our study provides a holistic view of the underlying molecular networks operating in the context of natural allergen exposure.

EGFR is a complex signaling pathway that can be activated by multiple ligands (e.g. amphiregulin, EGF, epiregulin, HB-EGF, TGF-a)^29^. Puddicombe *et al*. reported that EGFR was upregulated in the bronchial epithelium of patients with asthma and in particular severe asthma in comparison to healthy controls, and expression levels were correlated with sub-epithelial reticular membrane thickening^30^. Le Cras *et al*. reported that inhibition of EGFR signaling with a tyrosine kinase inhibitor reduced goblet cell hyperplasia, airway hyperreactivity and airway smooth muscle thickening in a chronic mouse model of HDM exposure^31^. The latter two phenotypes were also reduced by conditional transgenic expression of a dominant negative EGFR mutant in the lung epithelium. Together, these data suggest that upregulation of EGFR signaling in the context of HDM exposure plays a causal role in the development of asthma-related traits.

ERBB2 is an orphan receptor from the EGFR family. It lacks a ligand-binding domain and transduces signals by forming heterodimers with other ligand bound members of the EGF receptor family, including EGFR. Polosa *et al*. reported that ERBB2 expression was not different in bronchial epithelial cells from asthmatic subjects compared to healthy controls^32^. Song and Lee identified ERBB2 as an asthma susceptibility gene based on a pathways analysis of genome-wide single nucleotide polymorphism data^33^. Modena *et al*. studied coexpression networks in bronchial epithelial brushings from severe asthmatics and showed that ERBB2 was a major hub^34,35^. To date, the function of ERBB2 in asthma has not been investigated. Vermeer *et al*. reported that blockade of ERBB2 signaling in differentiated airway epithelial cells cultured at air-liquid interface reduced the number of ciliated epithelial cells^36^. Kettle *et al*. reported that blocking ERBB2 signaling *in vitro* attenuated neuregulin-induced upregulation of MUC5AC and MUC5B^37^. Notably, our network analysis showed that ERBB2 connects to anterior gradient 2 (AGR2) and ERBB2 has been shown to upregulate the transcription and secretion of AGR2^38,39^. AGR2 binds to immature MUC5AC in the endoplasmic reticulum, where it is thought to play a role in mucin folding. AGR2 deficient mice have profound defects in intestinal mucus production and reduced mucus production in the airways of allergen challenged mice^40,41^. It has also been shown that deficiency in the ERBB2 adapter protein ERBB2IP (also known as ERBB2 interacting protein or ERBIN), leads to deregulation of TGFB1 signaling, and this in turn is associated with type 2 activation^42^. In summary, upregulation of ERRB2 networks may influence asthma pathogenesis by modulating epithelial differentiation, mucus production and/or type 2 signaling.

E-cadherin (CDH1) is a cell adhesion molecule that forms adherence junctions between adjacent airway epithelial cells and maintains epithelial barrier integrity^43^. HDM disrupts epithelial barrier function by delocalizing E-cadherin and other junction molecules, and this is thought to enhance allergic sensitization and inflammation^44^. Polymorphisms in CDH1 are associated with airways remodeling and lung function decline, but only in those asthma patients using corticosteroids^45^. Dysregulation of CDH1 networks may impact on barrier function, inflammation, and airways remodeling.

IL-13 plays a central role in the pathogenesis of asthma by driving mucus production, airways hyper-responsiveness, and airways remodeling^24^. It is produced by Th2 and group 2 innate lymphoid cells (ILC2), and it can also be produced by macrophages^46,47^. IL-13 itself was not differentially expressed in HDM^S^ wheezers versus nonwheezers, however network analysis demonstrated that in wheezers it was connected to an extensive set of genes that have established roles in mouse models of allergic asthma. For instance, IL-33 stimulates the production of IL-5 and IL-13 by type 2 innate lymphoid cells and type 2 cells^48,49^, and in the presence of GM-CSF it can drive allergic inflammation at sub-threshold allergen doses^50^. In animal models, deficiency of multiple genes from the IL-13 network can impact on asthma-related traits, including allergic sensitization and/or inflammation (ALOX15^51^, CYBB^52^), and airways hyperresponsiveness and mucus production/goblet cell hyperplasia (POSTN^53^, SERPINB3/4^54^). Moreover, transgenic expression of SPDEF or FOXA3 leads to upregulation of pulmonary type 2 cytokines and increased goblet cell differentiation, eosinophilic inflammation, and airway hyperresponsiveness^55^. It is noteworthy, that whilst both IL-13 and EGFR ligands can induce the transcription of mucin genes, microarray profiling studies have shown that these pathways have largely independent effects on gene regulation in bronchial epithelial cells, and they play distinct roles in goblet cell metaplasia^40,56,57^. Many other pathways were also identified that are regulated by IL-13 and relevant to asthma pathogenesis (e.g. CCL17, CCL26, CTGF, FCER1A, KITLG, MUC2, NOS2, TLR3, Supplementary Table S6).

The CDHR3-associated coexpression module comprised genes expressed in ciliated epithelial cells that control mucociliary clearance. The primary function of cilia is to beat in a synchronous manner to clear mucus from the airways and into the pharynx. Thomas *et al*. reported that cilia beat frequency was decreased in patients with asthma, and severe asthmatics had abnormal ciliary orientation and microtubule defects^58^. Notably, employing network analysis we showed that CDHR3 was a highly ranked coexpression hub within this module. Further, Griggs *et al*. recently demonstrated that CDHR3 is exclusively expressed on ciliated airway epithelial cells and not on other epithelial cells (e.g. goblet and basal cells) consistent with our network analysis showing that CDHR3 is associated with ciliated epithelial cells^59^. Bonnelykke *et al*. reported that polymorphisms in CDHR3 were associated with recurrent, severe childhood asthma exacerbations^20^. Bochkov *et al*. showed that CDHR3 is the receptor for Rhinovirus-C^21^. Of note, CDHR3 was selected for these validation studies, because although it is a susceptibility gene for severe asthma exacerbations and a receptor for Rhinovirus-C, its endogenous cellular role is unknown. The other genes in this module have well-established roles in the function of cilia. For example, FOXJ1 is a transcription factor, which is essential for the formation of motile cilia; PIFO controls cilia retraction; and dyneins (e.g. DNAH5, DNAH9) are the motor proteins that mediate ciliary beating. We examined CDHR3 protein expression in bronchial epithelial cells, and we demonstrated that expression was localized to the apical surface of columnar epithelial cells and was increased in HDM sensitized atopics with asthma compared to nonatopic controls. Ross *et al*. reported that CDHR3 was highly upregulated during mucociliary differentiation of human airway epithelial cells^60^. Detailed mechanistic studies will be required to dissect the role of CDHR3 in ciliated epithelial cells.

The findings above highlight candidate pathways underlying airways inflammation and airway remodeling in the pathogenesis of asthma/wheeze, and our computational analysis which combines data-driven coexpression analysis together with mechanistic data from prior studies to infer network structure suggests that under baseline conditions during ongoing aeroallergen exposure, these pathways are intertwined and work in concert. Moreover, asthma is a highly heterogeneous disease, and the contribution of type 2 inflammation versus airways remodeling is likely to be highly variable from subject to subject^7,61^. Our study also represents a single snapshot in time, and thus the stability of these phenotypes on repeated sampling is unknown. In particular, superimposition of respiratory infection upon this background of persistent aeroallergen exposure, which is known to precipitate acute exacerbations and to accelerate lung function decline in asthmatics^62^, may markedly alter the interactions between and/or the relative contributions of these pathways to the overall process. Follow-up studies in larger populations will be required to further elucidate these issues.

This exploratory study has limitations that should be acknowledged. The molecular profiling studies were based on a heterogeneous cell population, and the pathways we identified were mainly associated with airway epithelial cells, which represent a minority population in sputum. The proportion of epithelial cells in the sputum samples did not differ between the HDM^S^ subjects with or without wheeze, but it was not possible to differentiate between ciliated and non-ciliated epithelial cells. Future studies employing single cell RNA-Seq profiling will provide more detailed information on the role of individual cell types in this phenotype. The differentially expressed genes we identified were related to proportions of eosinophils, but this was not surprising given that eosinophils were increased in HDM^S^ wheezers and are known to correlate with type 2-associated inflammation^61^. We observed asymmetry in the number of up and downregulated genes in our analyses. Previous studies suggest this may occur when RUV adjustment is employed to remove the effect of unwanted variation in the data, but this adjustment was necessary to obtain a uniform p-value distribution^63^. Prior knowledge was employed to reconstruct the gene networks, which relies on data derived from experimental settings that may be far removed from the current study, and this is oversimplified given that genes can function in a context specific manner. Follow-up mechanistic studies will therefore be required to elucidate the specific cellular and molecular mechanisms involved. Additionally, hyperosmotic stress effects on epithelial cells resulting from mannitol treatment may have influenced the gene expression profiles detected, but this is unlikely to explain the main differences between those with/without wheeze as the treatment was standardized between the groups. Notwithstanding these caveats, our findings are consistent with the general hypothesis that progression from subclinical responsiveness to aeroallergen exposure in atopic asthmatics to expression of the persistent wheezing phenotype involves the establishment of coexpression networks linking type 2 effector pathways in immune cells recruited to the airway surface with genes expressed in adjacent epithelial cells that have been implicated in myriad asthma-relevant functions, including mucosal barrier integrity, mucus production, tissue remodeling, responsiveness to irritants, and (exemplified by IL-33) intensification of aeroallergen-specific type 2 immunity. Targeting drug development programs specifically at these chronic mechanisms, as opposed to simply those that are triggered during acute exacerbation events, may provide improved therapeutics for prevention of asthma progression in atopics who represent the segment of the population at greatest risk of this disease.

## Methods

### Study population

This study was conducted within the 22-year follow-up of an unselected longitudinal birth cohort recruited in Perth, Western Australia, namely the Western Australia Pregnancy Cohort (Raine study, refs^11,25^). The 22-year follow-up included 1234 active participants. All aspects of the study were approved by the University of Western Australia Human Ethics Committee (ethics number: RA/4/1/5202), carried out in accordance with the University guidelines and regulations, and participants provided written informed consent. Subjects were selected for case/control studies based on their clinical characteristics and the availability of high quality sputum samples (see below). Participants were classed as having current wheeze if they indicated in the 22-year follow-up questionnaire that they had wheezed in the past 12 months. Current asthma was defined as a positive doctor diagnosis of asthma ever, in addition to both wheeze and asthma medication use in the past 12 months^12^. Atopy was defined by skin prick test wheal ≥3 mm for the common allergens as outlined in the online supplement. Four clinical groups were defined; (i) HDM sensitized atopics (SPT ≥ 3.0 mm) with current wheeze during previous 12mths, with or without a physician diagnosis of “asthma ever” (HDM^S^ wheezers, n = 16); (ii) HDM sensitized atopics without current asthma or wheeze (HDM^S^ nonwheezers, n = 24); (iii) nonatopics with current asthma and/or wheeze (nonatopic wheezers, n = 7); (iv) nonatopics without current asthma or wheeze (nonatopic controls, n = 21).

### Sputum induction and processing

Participants were required to cease mediation use for a period of 72 h prior to respiratory assessment and sputum collection. Induced sputum was obtained after mannitol inhalation challenge^64^. The samples were stored at 4 °C for up to 2 hours prior to processing. Sputum was processed (see the online data supplement) by selection and subsequent disruption of mucus plugs with forceps to minimize contamination with saliva^65^.

### Transcriptome profiling by RNA-Seq

Total RNA was extracted from good quality sputum (cell viability > 48%; squamous cell contamination <32%; RNA integrity number >6) employing TRIzol (Ambion) followed by RNeasy MinElute (QIAgen). The mean ± sd RNA integrity number was 7.6 ± 1.0 as assessed on the bioanalzyer (Agilent). RNA samples were shipped on dry ice to the Australian Genome Research Facility for library preparation (TruSeq Stranded mRNA Library Prep Kit, Illumina) and sequencing (Illumina HiSeq. 2500, 50-bp single-end reads, v4 chemistry). The raw data are available at the NCBI Short Read Archive (accession; SRP057350).

### RNA-Seq data analysis

The quality of the RNA-Seq data was assessed with the Bioconductor package Rqc (Supplementary Fig. S2). Reads were aligned to the reference genome (hg19) using Subread, and summarized at the gene-level using featureCounts^66^. Genes with less than 300 total counts were removed from the analysis. Differentially expressed genes were identified employing Empirical analysis of digital gene expression data in R (EdgeR) with Benjamini-Hochberg False Discovery Rate (FDR) control for multiple testing^67^. The analysis was adjusted using the Remove Unwanted Variation (RUV) algorithm^68^ to minimize the potential for biological and/or technical variation to impact on the analysis (Fig. S3). All factor levels were analysed in a single model, with or without adjustment for unwanted variation and additional covariates.

A coexpression network was constructed employing the weighted gene coexpression network analysis (WGCNA) algorithm^16^. Prior to network analysis, the count data was transformed using the variance stabilizing transformation algorithm^67^. Modules associated with clinical traits were identified by plotting the −log10 p-values from the edgeR analysis on a module-by-module basis. The wiring diagram of selected gene networks was reconstructed employing two different methods. The first method utilized “prior knowledge” comprising experimentally supported molecular relationships based on data from the Ingenuity Systems KnowledgeBase (www.ingenuity.com)^16^. The second method utilized unbiased connectivity patterns derived from WGCNA, and the network was visualized using VisANT^19^. Biological pathways and functions enriched in the data were identified with MSigDb and Enrichr^22,69^. Upstream regulator analysis was performed using Ingenuity systems software, and the threshold for statistical significance was *p*-value < 0.01 and absolute activation Z-score > 2.0^23^.

### Immunostaining

Primary bronchial epithelial cells were obtained from 13 healthy nonatopic children and 12 atopic asthmatic children with HDM allergy who were undergoing elective surgery for non-respiratory related conditions. Samples were obtained under a separate study that was approved by the Princess Margaret Hospital for Children’s Human Ethics Committee (937EP), and all methods were performed in accordance with the relevant guidelines and regulations. Written consent was obtained from each participant’s legal guardian after being fully informed about the nature and purpose of the study. Cytospins were prepared and stained for CDHR3, EGFR, ERBB2 and DAPI using methods previously described (see online supplementary methods).

### Preprint Server

This work has been posted as a preprint on the bioRxiv server^70^.

## Electronic supplementary material

Supplementary Information

Supplementary Tables S1–11
