# Supplementary material for: Persistent activation of interlinked type 2 airway epithelial gene networks in sputum-derived cells from aeroallergen-sensitized symptomatic asthmatics
Source: Sci Rep. 2018 Jan 24;8:1511. doi: 10.1038/s41598-018-19837-6 (PMC5784090; doi:10.1038/s41598-018-19837-6)
Supplement: Supplementary file 1 — Supplementary Information [file 41598_2018_19837_MOESM1_ESM.doc]

**Online Data Supplement**

**Persistent activation of interlinked type 2 airway epithelial gene networks in sputum-derived cells from aeroallergen-sensitized symptomatic asthmatics**

**Authors**

Anya C. Jones^, MSc†§, Niamh M. Troy^, BSc†, Elisha White, MHltSci†, Elysia M. Hollams, PhD†, Alexander M. Gout, PhD†, Kak-Ming Ling, BSc†, Anthony Kicic, PhD†‡§ll, Stephen M. Stick, MD PhD†‡§ll, Peter D Sly, MD DSc∆, Patrick G Holt, Dsc FAA†∆, Graham L Hall, PhD†¶#, Anthony Bosco, PhD†

**Affiliations**

† Telethon Kids Institute, The University of Western Australia;

§ School of Paediatrics and Child Health, The University of Western Australia, Western Australia;

‡ Department of Respiratory Medicine, Princess Margaret Hospital for Children, Western Australia;

ll Centre for Cell Therapy and Regenerative Medicine, School of Medicine and Pharmacology, The University of Western Australia, Western Australia;

∆ Child Health Research Centre, The University of Queensland, Brisbane, Australia.

¶ School of Physiotherapy and Exercise Science, Curtin University

# Centre of Child Health Research, UWA

**Author contributions**

^ These authors contributed equally to this work. Conception and design of research: AB, GLH, PGH; Acquisition of data: ACJ, NMT, EW, KML, AK, SMS, PDS; Data analysis: ACJ, NMT, EW, EMH, AMG, KML, AK, SMS, AB; Drafting the manuscript for important intellectual content: ACJ, NMT, PGH, AB; Approval of final version of manuscript: ACJ, NMT, EW, EMH, AMG, KML, AK, PDS, PGH, GLH, AB.

Table of Contents

[Study population 2](#__RefHeading___Toc494980548)

[Sputum induction and processing 2](#__RefHeading___Toc494980549)

[Transcriptome profiling by RNA-Seq 4](#__RefHeading___Toc494980550)

[RNA-Seq data analysis 4](#__RefHeading___Toc494980551)

[Microarray data analysis (replication cohorts) 5](#__RefHeading___Toc494980552)

[Immunostaining 6](#__RefHeading___Toc494980553)

[Figure S1. Flow diagram illustrating the selection of sputum samples. 8](#__RefHeading___Toc494980554)

[Figure S2. Quality assessment of the RNA-Seq data. 9](#__RefHeading___Toc494980555)

[Figure S3. Relative log expression (RLE) plot. 10](#__RefHeading___Toc494980556)

[Figure S4. Identification of modules associated with HDM sensitization and asthma. 11](#__RefHeading___Toc494980557)

[Figure S5. Gene network diagram for module “P”. 12](#__RefHeading___Toc494980558)

[Figure S6. Gene network diagram for module “Q”. 13](#__RefHeading___Toc494980559)

[Figure S7. Cluster dendrogram to quantify the correlation between modules. 14](#__RefHeading___Toc494980560)

[Figure S8. The reconstructed gene networks of the merged modules “P” and “Q”. 15](#__RefHeading___Toc494980561)

[Figure S9. Identification of wheeze-associated modules with or without adjustment for covariates. 16](#__RefHeading___Toc494980563)

[Figure S10. Replication of the CDHR3 module (module “A”) in two independent asthmatic data sets. 17](#__RefHeading___Toc494980564)

[Figure S11. Immunostaining of CDHR3 (in green) and DAPI (in blue) of bronchial epithelial cells of nonatopic controls (n=8) and atopic asthmatics (n=8). 18](#__RefHeading___Toc494980566)

[Figure S12. Immunostaining of EGFR (in red) and DAPI (in blue) of bronchial epithelial cells of nonatopic controls (n=5) and atopic asthmatics (n=5). 19](#__RefHeading___Toc494980569)

[Figure S13. Immunostaining of ERBB2 (in red) and DAPI (in blue) of bronchial epithelial cells of nonatopic controls (n=5) and atopic asthmatics (n=5). 20](#__RefHeading___Toc494980571)

[References 21](#__RefHeading___Toc494980572)

# Study population

This study was conducted within the 22-year follow-up of an unselected longitudinal birth cohort recruited in Perth, the Western Australia Pregnancy Cohort (Raine study, [^1^](#_ENREF_1)). The study included 2868 infants at birth, and follow-up visits at 1, 2, 3, 6, 8, 10, 14, 17, 18 and 20 years of age [^2-4^](#_ENREF_2). Of the 1,234 study participants, 68 high-quality sputum samples were sequenced based on RNA quality (Fig S1). The study participants completed a questionnaire pertaining to respiratory health, they underwent skin prick testing, baseline spirometry and mannitol challenge tests, and exhaled nitric oxide measurement. The participants were required to withhold asthma medications and antihistamines for 72 hours prior to testing, and compliance was verified by questioning on the day of testing. Participants were classed as having current wheeze if they indicated in the 22-year follow-up questionnaire that they had wheezed in the past 12 months. Current asthma was defined as a positive doctor diagnosis of asthma ever, in addition to both wheeze and asthma medication use in the past 12 months. Atopy was defined by skin prick test wheal ≥3mm for the following allergens: House dust mite *Dermatophagoides pteronyssinus*; House dust mite *Dermatophagoides farinae;* grass mix, grass pollen, dog hair, cat hair, cockroach, mold mix, cow’s milk, egg white.

# Sputum induction and processing

Participants were required to cease mediation use for a period of 72 h prior to respiratory assessment and sputum collection, and were questioned to verify medication withholding on the day of testing. Induced sputum was obtained after mannitol inhalation challenge based on the approach first reported by Wood and co-workers [^5^](#_ENREF_5). Briefly, an inhaled mannitol challenge test was performed according to the manufacturer’s recommendation (Pharmaxis Ltd, Frenchs Forest, NSW, Australia). Baseline spirometry was performed (nSpire Health KOKO PFT spirometer) and FEV1 measurements during the challenge test were performed according current spirometry guidelines [^6^](#_ENREF_6). After each inhalation the participant was encouraged to cough and any sputum produced was collected. The challenge test continued until the final dose (635 mg) or if there was a 15% drop in FEV1 from baseline or a 10% drop between doses occurred (indicating a positive test). Baseline spirometry data are reported in absolute and predicted terms. Predicted lung function was based on the 2012 Global Lung Function Initiative equations [^7^](#_ENREF_7) the validity of which has been confirmed in an Australasian population [^8^](#_ENREF_8). Exhaled nitric oxide measurement was performed according to international guidelines. Briefly, participants inhaled NO-free air to total lung capacity and immediately exhaled through a mouthpiece and a constant flow of 50 mL/s. A minimum of 3 acceptable measurements that varied by less than 10% were obtained and the average eNO in parts per billion (ppb) reported [^9^](#_ENREF_9).

Mucus plugs were selected with forceps and disrupted in AIM-V media (Life Technologies) containing 10% (v/v) sputolysin (Calbiochem) and incubated for 10 min at 37°C with intermittent vigorous pipetting. The released cells were centrifuged, and a cytospin slide was prepared for blinded differential cell counting. Inflammatory cell percentages were reported as percentages of all cells including squamous cells. Epithelial cell frequencies were calculated of total cells including squamous cells. The remaining cells were stabilized in RNAprotect cell reagent (QIAgen) and stored at -80 °C for molecular profiling studies.

# Transcriptome profiling by RNA-Seq

Total RNA was extracted from sputum employing TRIzol (Ambion) followed by RNeasy MinElute (QIAgen). The total RNA samples were shipped on dry ice to the Australian Genome Research Facility for library preparation (TruSeq Stranded mRNA Library Prep Kit, Illumina) and sequencing (Illumina HiSeq2500, 50-bp single-end reads, v4 chemistry). Approximately 25 million reads were generated from each sample. The raw sequencing data are available at the NCBI Short Read Archive under accession SRP057350.

# RNA-Seq data analysis

The quality of the RNA-Seq data was assessed with the Bioconductor package Rqc [^10^](#_ENREF_10) (Fig. S2). Sequencing reads were aligned to the reference genome (hg19) using Subread [^11^](#_ENREF_11). Reads were counted and summarized at the gene-level using featureCounts. Genes with less than 300 counts in total were removed from the analysis. Differentially expressed genes were identified employing Empirical analysis of digital gene expression data in R (EdgeR) with Benjamini-Hochberg False Discovery Rate (FDR) control for multiple testing [^12^](#_ENREF_12) (Tables S3-5). EdgeR is based on the negative binomial distribution, which is an extension of the Poisson distribution. EdgeR employs empirical Bayes methods to estimate gene-specific biological variation and moderate these estimates towards a trended mean [^12^](#_ENREF_12). The analysis was adjusted for latent variation using the Remove Unwanted Variation (RUV) algorithm [^13^](#_ENREF_13) (Fig. S3). All factor levels were analysed in a single model, with or without adjustment for unwanted variation and additional covariates.

A coexpression network was constructed from the filtered RNA-Seq data (genes with < 300 total counts were removed from the analysis; 14,833 genes remained) employing the weighted gene coexpression network analysis (WGCNA) algorithm (parameters; power = 6, Pearson correlation, minimum module size = 100, merge cut height = 0.1, pamstage=TRUE) . Prior to network analysis, the count data was transformed using the variance stabilizing transformation algorithm from the DESeq2 package [^16^](#_ENREF_16). The modules were analysed by principal component analysis followed by cluster analysis to quantify their overall correlation. Modules associated with clinical traits were identified by plotting the –log10 p-values derived from an edgeR analysis on a module-by-module basis (Figures S4A, S4B, S4C). Data analysis code is not available for the public but can be released upon request.

The wiring diagram of modules selected for further study was reconstructed employing experimentally supported molecular relationships from the Ingenuity Systems KnowledgeBase (Figures S5, S6). Principal component analysis of the modules suggested that the modules in Fig S5 and S6 were highly correlated (Pearson correlation: 0.897, P-value = 4.441 x 10^-15^) (Figure S7). We therefore merged them and reconstructed the network (Figure S8).

# Microarray data analysis (replication cohorts)

Two microarray data sets were downloaded from the Gene Expression Omnibus. The first data set consisted of asthmatic bronchial epithelial brushings (GEO41863), and the second data set was asthmatic sputum (GEO76226). The quality of the raw data was assessed utilising the R package arrayQualityMetrics [^18^](#_ENREF_18). Raw expression values were pre-processed with the robust multi-array average (RMA) algorithm [^18^](#_ENREF_18), employing updated custom chip description files (custom CDFs [^19^](#_ENREF_19)). Highly stringent principal component analysis-based filtering was employed and low quality probe sets were removed [^20^](#_ENREF_20). A coexpression network was constructed for each dataset employing WGCNA, and pathways analysis was performed using Enrichr [^21^](#_ENREF_21).

# Immunostaining

Primary bronchial epithelial cells were obtained from 13 healthy nonatopic children and 12 atopic asthmatic children with house dust mite allergy who were undergoing elective surgery for non-respiratory related conditions. The demographics of the pediatric study cohort are presented in Table S11. Asthma was defined as physician-diagnosed asthma together with documented wheeze by a physician in the previous 12 months. Atopy was determined by a positive radioallergosorbant (RAST) result to a panel of common allergens including; house dust mite, grass pollens, milk, mould, peanut, egg white and animal dander. All children with asthma had mild disease, such that none were being treated with inhaled or oral glucocorticosteroids.

Cytospins of ex vivo cells derived from airway sampling were initially created and stained for CDHR3 and DAPI using methods previously described [^22^](#_ENREF_22). Briefly, cells were incubated in a solution of 0.5% Sudan Black B in 70% ethanol to block auto-fluorescence. Cells were then washed in PBS and antigen retrieval performed by incubation with proteinase K. Cells were washed in PBS containing (0.1% v/v) saponin and blocked in 5%BSA, 10%FBS (v/v), 0.1% Triton X-100 in 1x PBS/saponin solution prior to incubation with polycloncal rabbit anti-human CDHR3 antibody (Novus Biologicals). Cells were washed again in PBS/saponin and incubated with AlexaFluor 488 (Life Technologies), mononuclear rabbit anti-human EGFR antibody [EP38Y] (Abcam) and monoclonal rabbit anti-ERBB2 antibody [EP1045Y] (Abcam). Cells were washed again in PBS/saponin and incubated with goat anti-rabbit IgG (H+L) AlexaFluor 488 (Life Technologies) for CDHR3 or with goat anti-rabbit IgG (H+L) AlexaFluor 568 (Life Technologies) for EGFR and ERBB2. Finally, nucleic acids were stained using DAPI (Sigma) and signals were then visualized using Nikon Eclipse Ti fluorescent microscope. All quantifications were performed using ImageJ software as previously described [^23^](#_ENREF_23).

Figure S1. Flow diagram illustrating the selection of sputum samples.

68 samples were sequenced on the bases of sputum and RNA quality.

Figure S2. Quality assessment of the RNA-Seq data.

A) Percentage of reads exceeding quality scores; B) Percent GC content for each sequencing cycle. This analysis was based on the R/Bioconductor package Rqc (7).


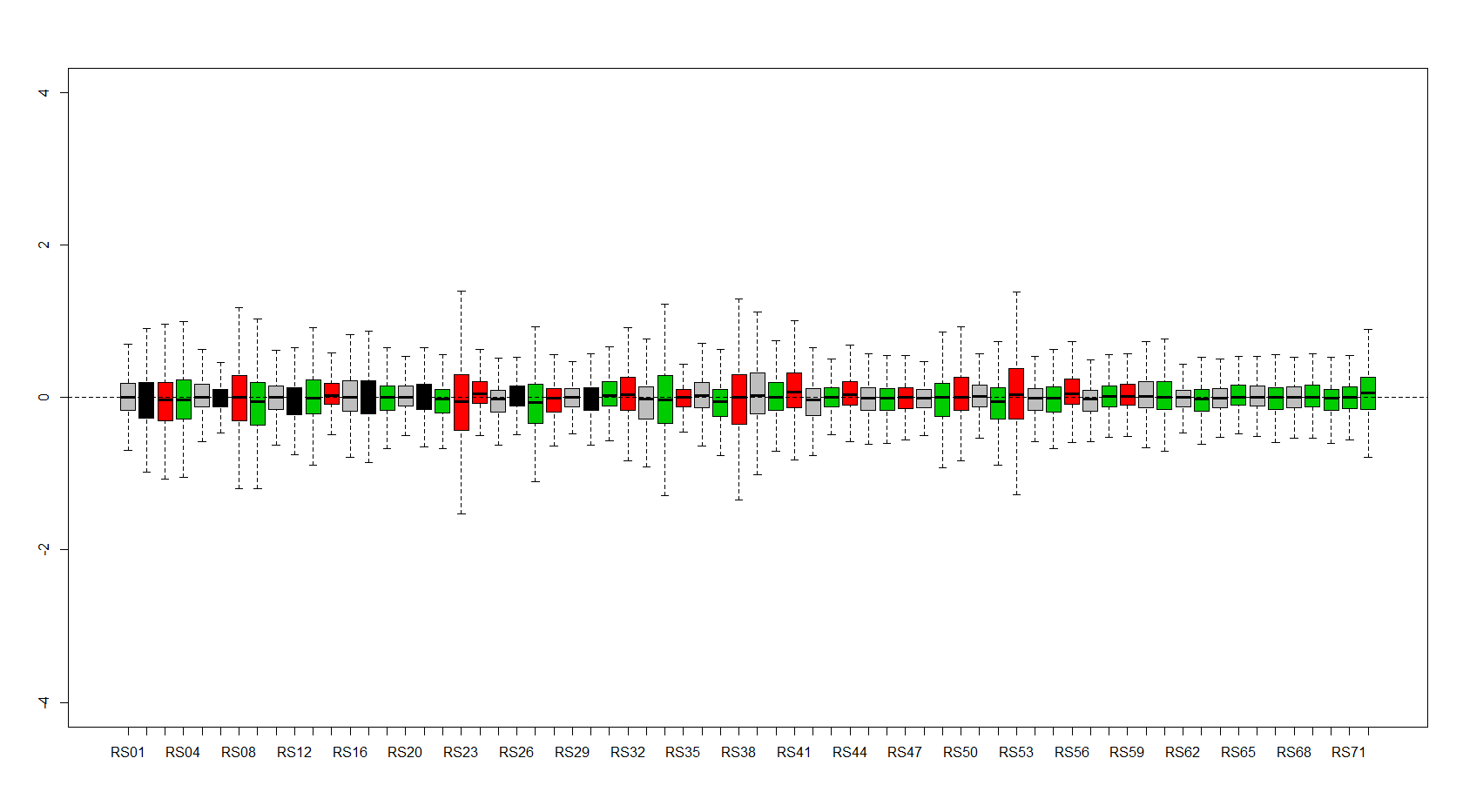


Figure S3. Relative log expression (RLE) plot.

The data reflect the log-ratios of the read counts of each sample with respect to the median of all samples after normalization with the remove unwanted variation algorithm (RUV) (E10). Grey = nonatopic controls; Black = nonatopic wheezers; Green = HDM^S^ nonwheezers; Red = HDM^S^ wheezers.


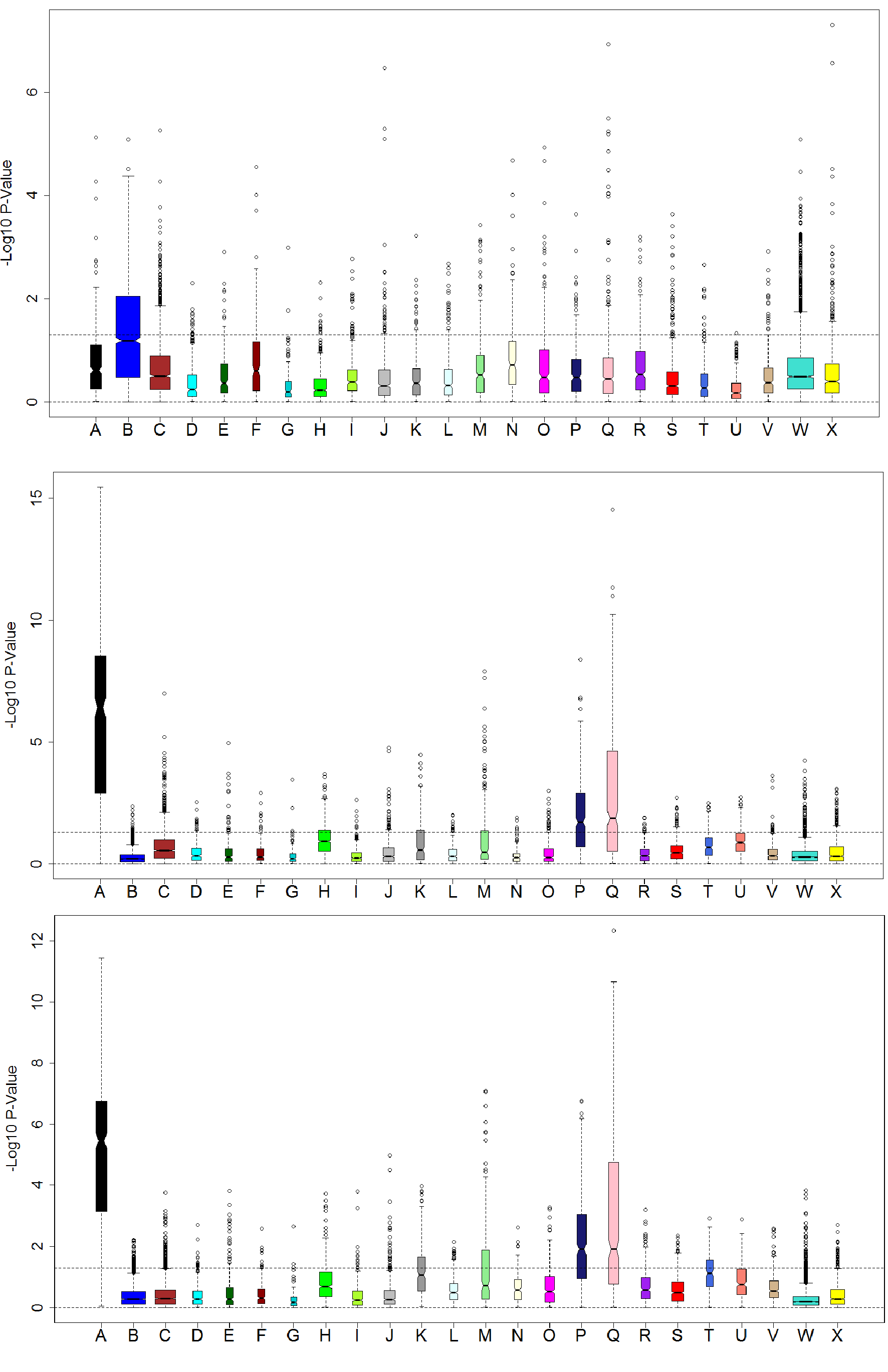
Figure S4. Identification of modules associated with HDM sensitization and asthma.

**A**

**B**

**C**

Gene network patterns were compared between A) HDM**^S^** nonwheezers versus nonatopic controls; B) HDM**^S^** wheezers versus nonatopic controls; (C) HDM**^S^** wheezers versus HDM**^S^** nonwheezers; FDR=0.0002 (Module A), FDR=0.1606 (Module P) and FDR=0.1589 (Module Q). The data represent the –log10 p-values derived from an edgeR analysis plotted as box plots on a module-by-module basis.


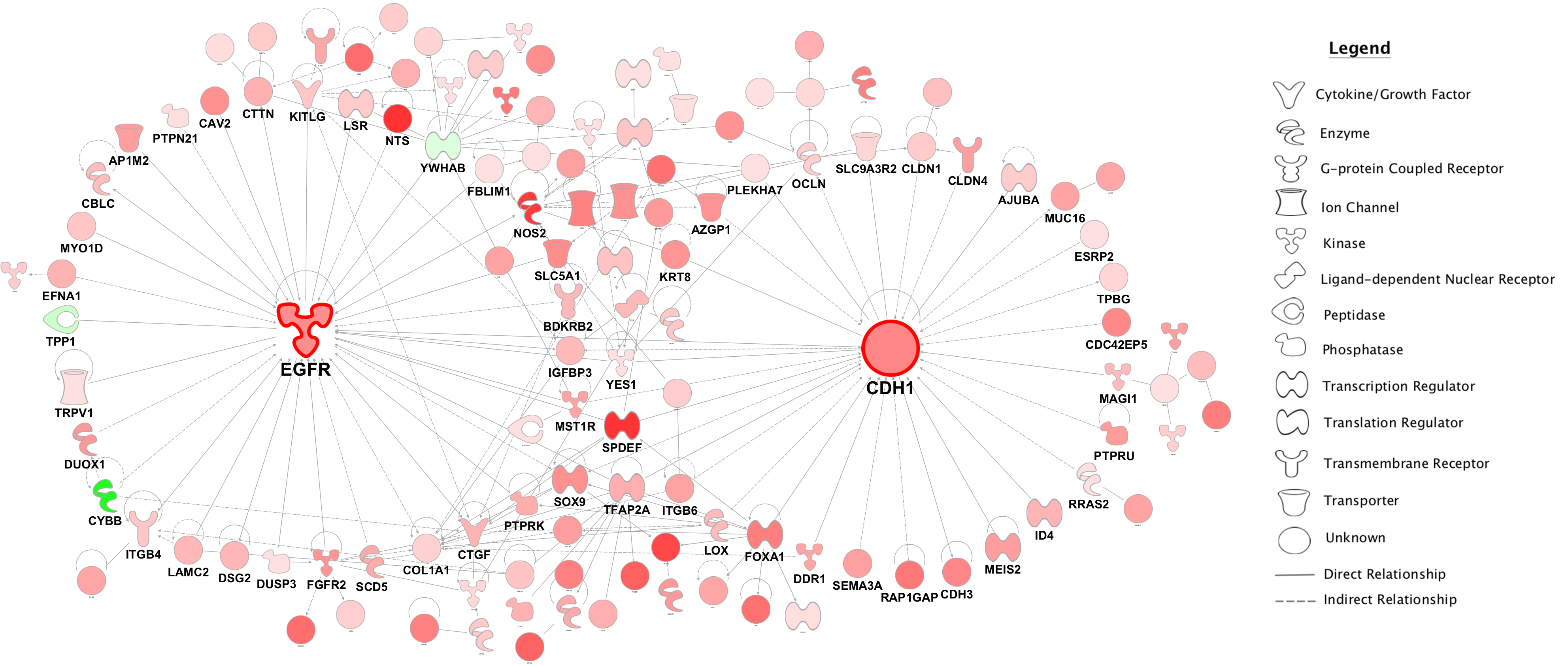


Figure S5. Gene network diagram for module “P”.

The gene network was reconstructed employing experimental data from prior studies. Red denotes upregulation, green denotes downregulation. The network was drawn with Ingenuity software (14).


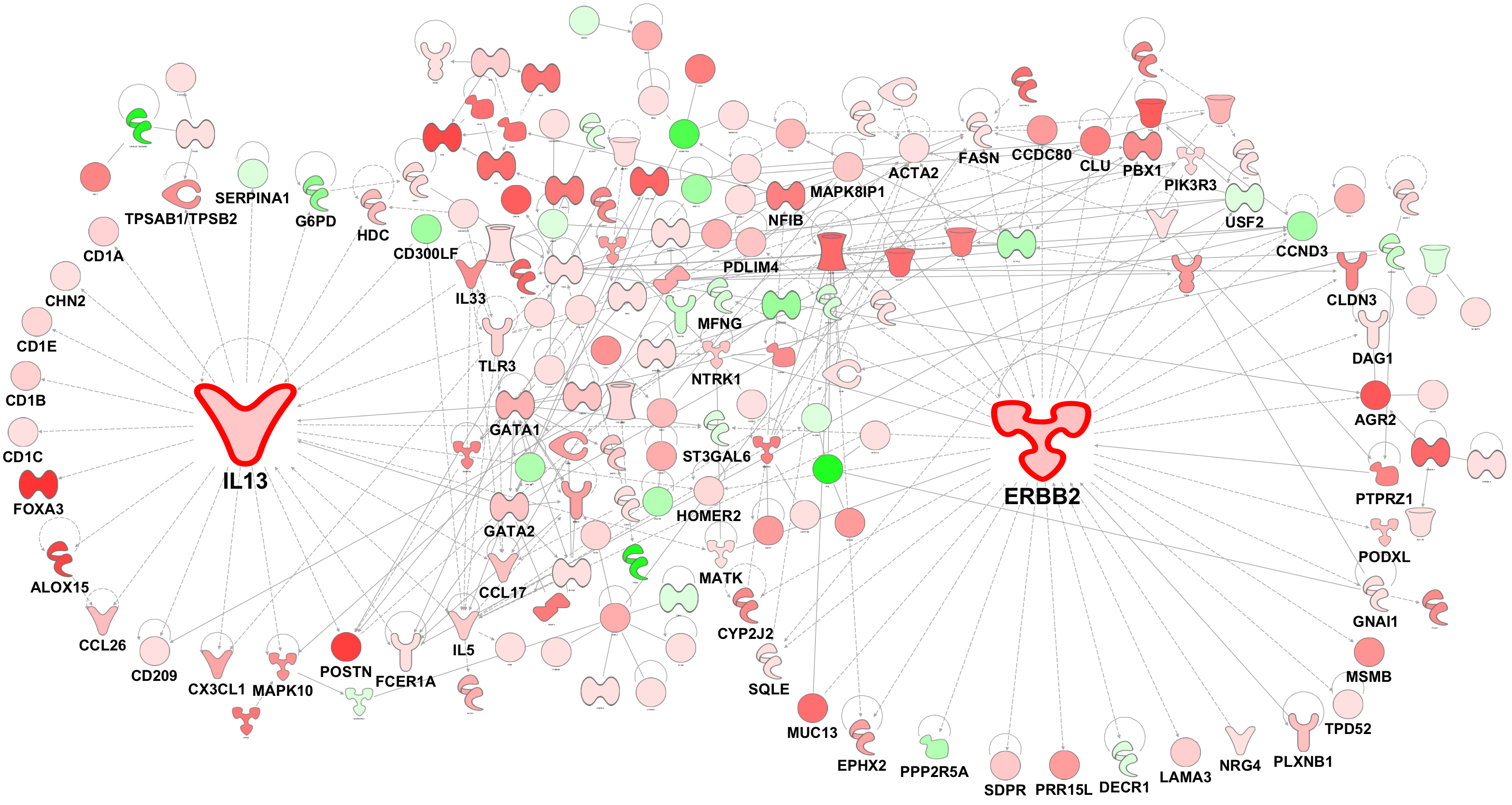


Figure S6. Gene network diagram for module “Q”.

The gene network was reconstructed employing experimental data from prior studies. Red denotes upregulation, green denotes downregulation. The network was drawn with Ingenuity software (14).


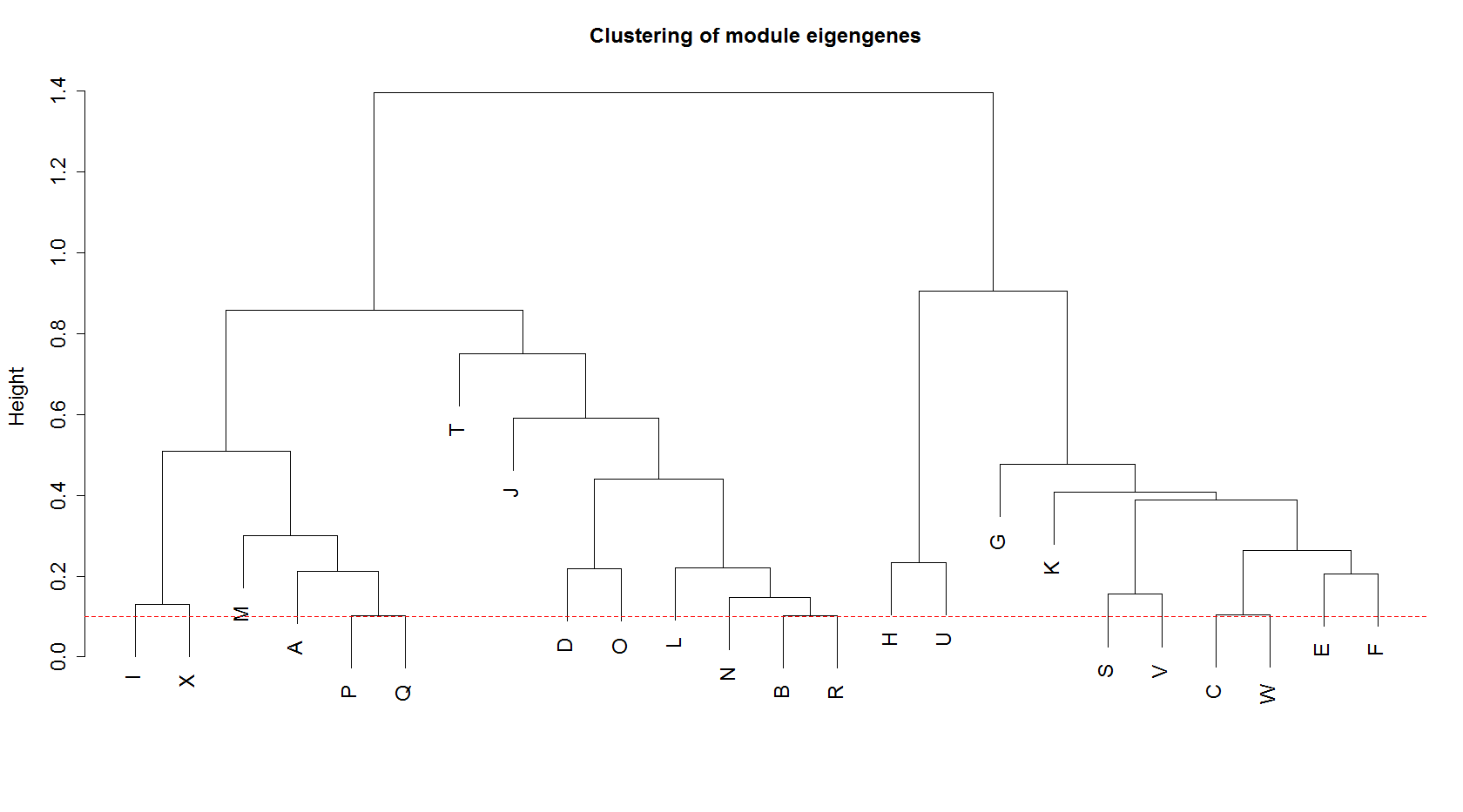


Figure S7. Cluster dendrogram to quantify the correlation between modules.

The overall expression of each module was summarized using principal component analysis, and the first principal component for each module (module eigengene) was correlated, subtracted from 1 to define a distance measure, and analyzed by hierarchical clustering. Note that modules “P” and “Q” were highly correlated (Pearson correlation: 0.897, P-value = 4.441 x 10^-15^); and therefore these modules were merged.


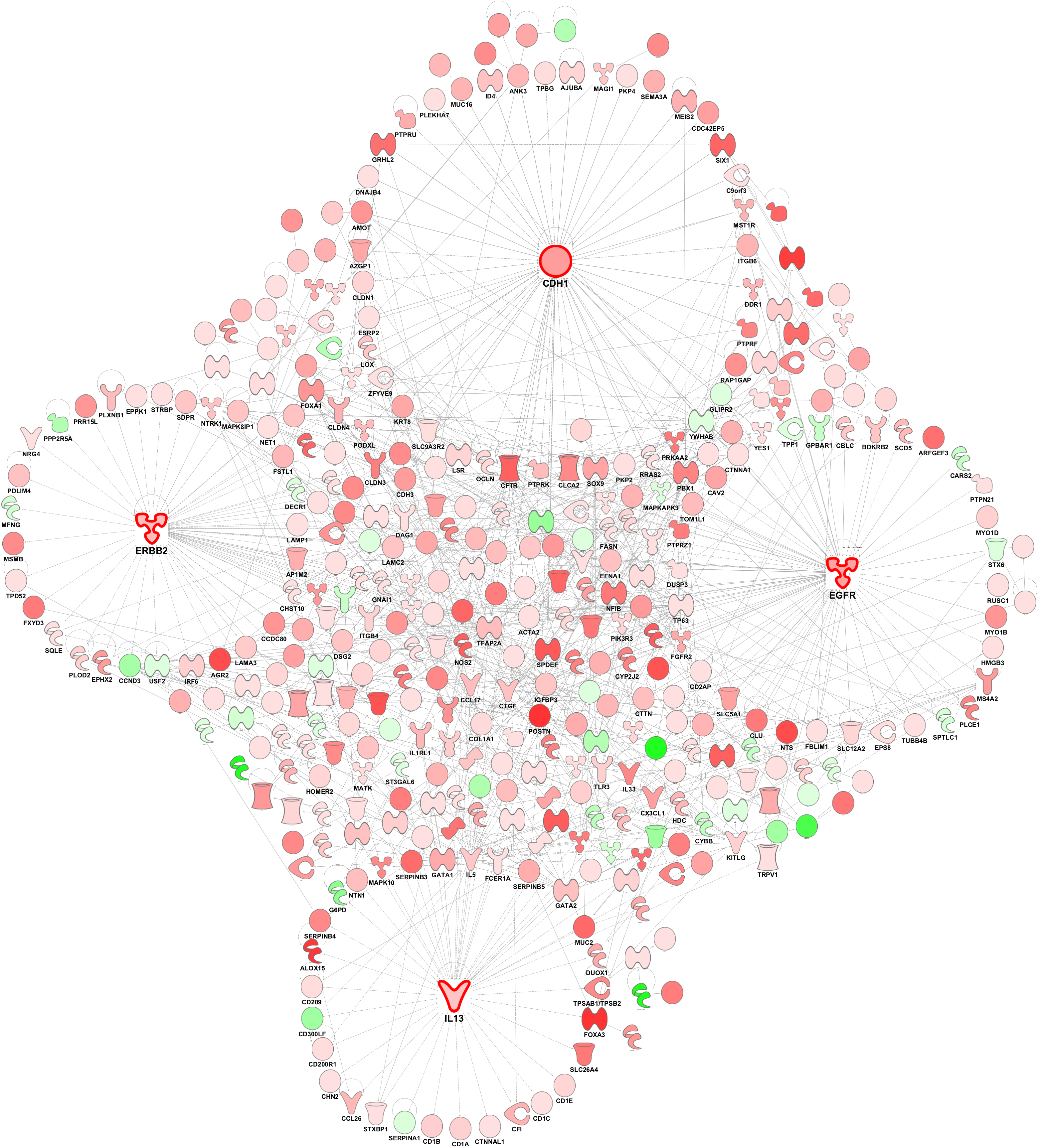


Figure S8. The reconstructed gene networks of the merged modules “P” and “Q”.

The gene network was reconstructed employing experimental data from prior studies. Red denotes upregulation, green denotes downregulation. The network was drawn with Ingenuity software (14).


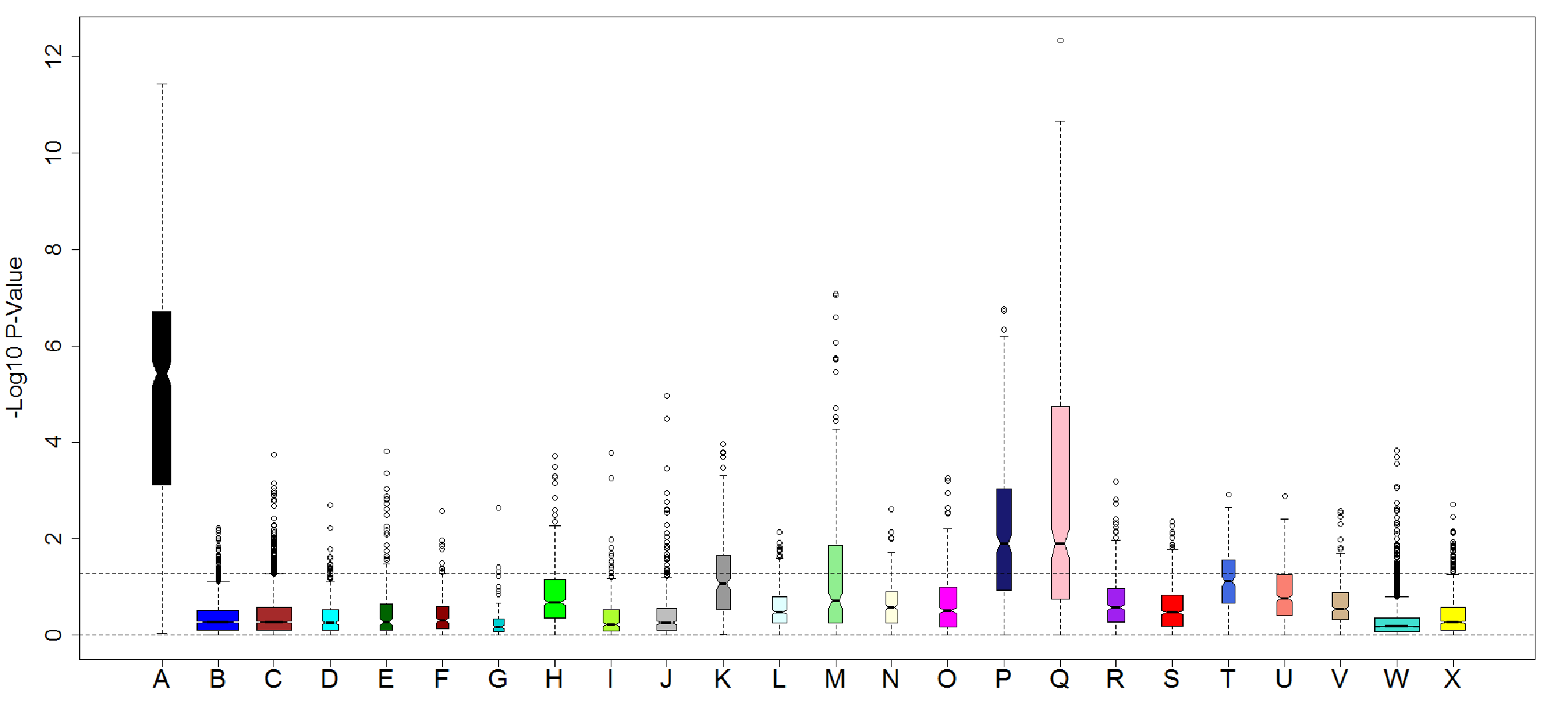

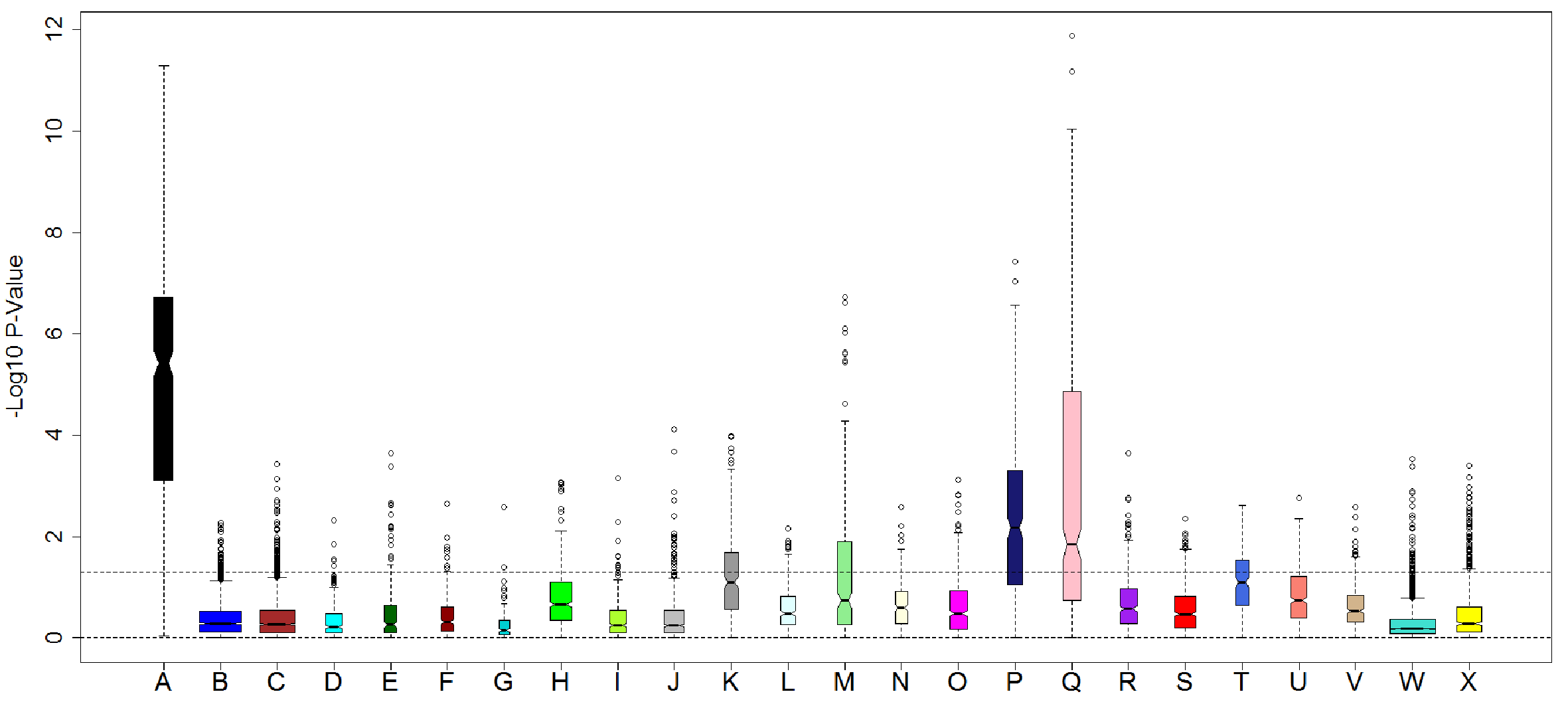

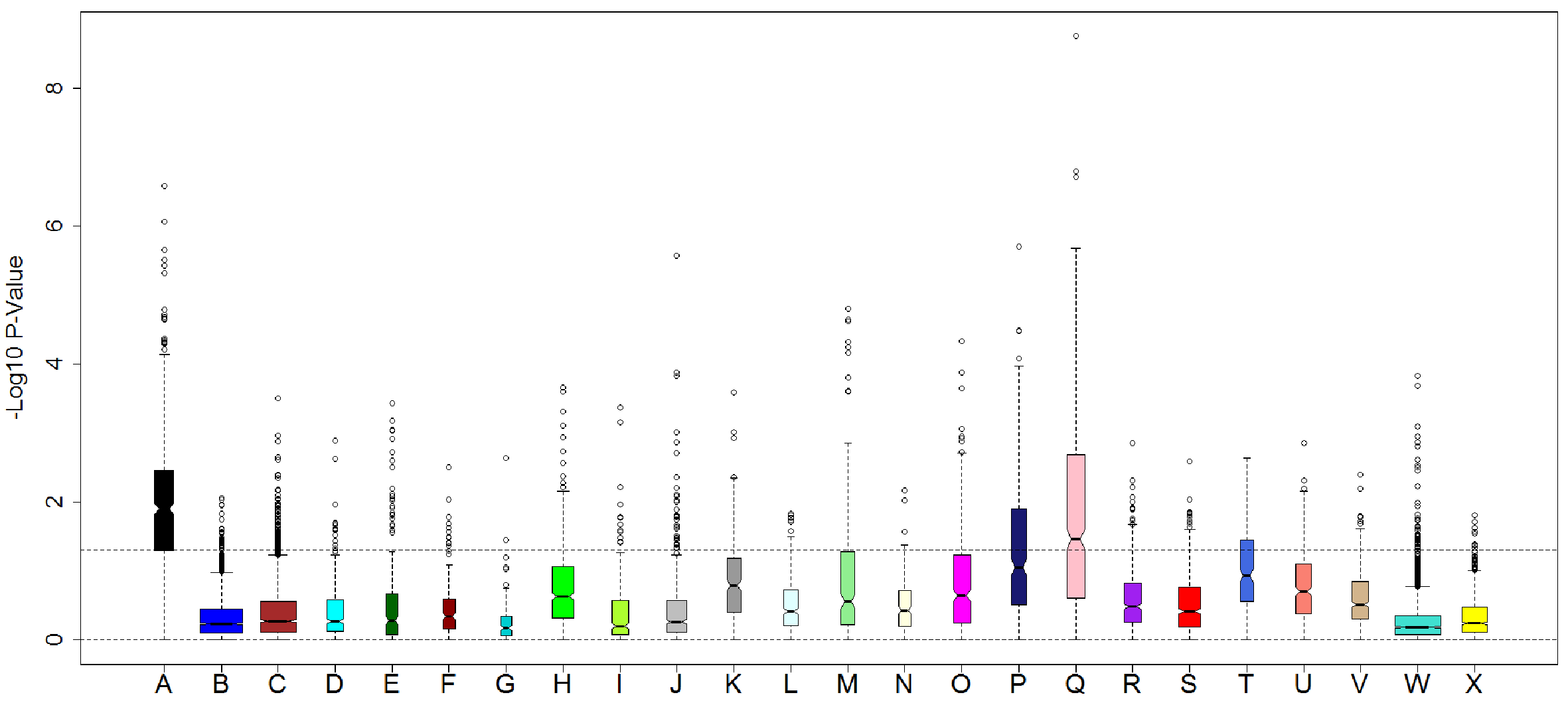

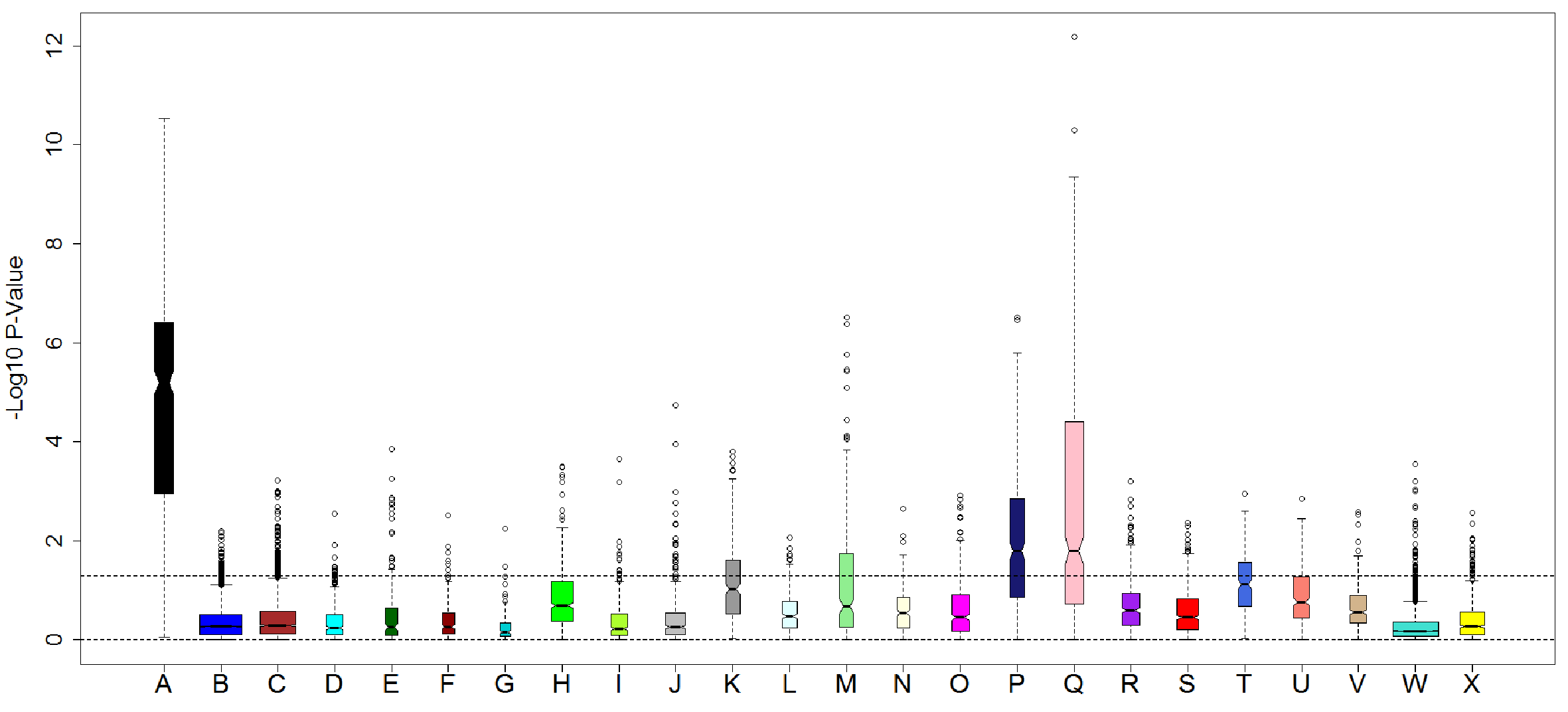


A)

D)

B)

C)

Figure S9. Identification of wheeze-associated modules with or without adjustment for covariates.

Data was analyzed with edgeR/RUVSeq, adjusted for: a) unadjusted, b) the proportions of squamous cells, c) the proportions of eosinophils, and d) smoking.

Figure S10. Replication of the CDHR3 module (module “A”) in two independent asthmatic data sets.

Network analysis (WGCNA) was carried out on two independent datasets to determine if the CDHR3-associated module was reproducible; In both data sets [a) asthmatic bronchial epithelial brushings GSE76226, b) asthmatic sputum (GSE41863)], the genes from the CDHR3 module were tightly clustered, as illustrated by the blue banding pattern. Moreover, CDHR3 was a highly ranked coexpression hub (rank 18/1750, rank 12/271) in each data set.


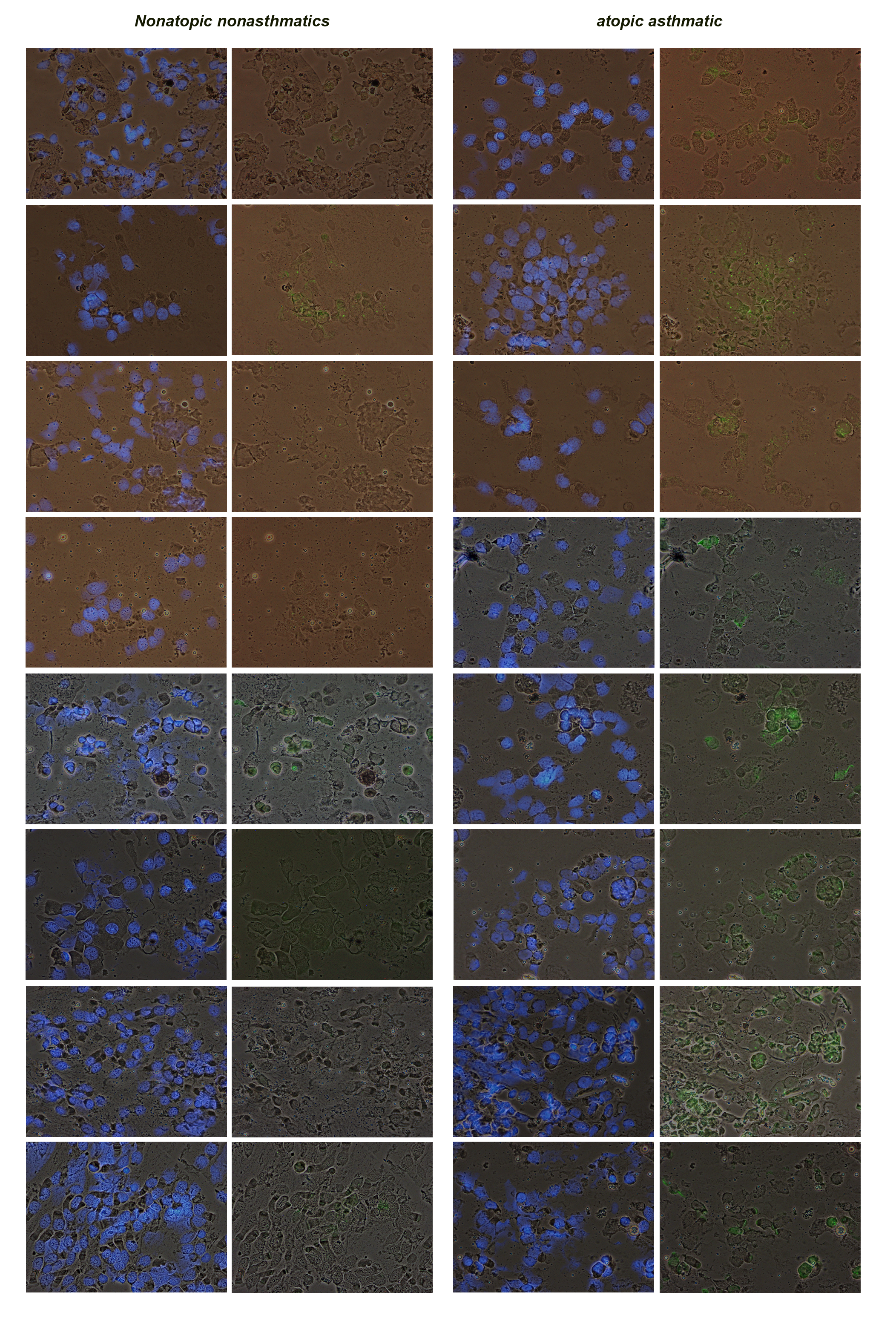


**nonatopic control**

**atopic asthmatic**

**CDHR3**

Figure S11. Immunostaining of CDHR3 (in green) and DAPI (in blue) of bronchial epithelial cells of nonatopic controls (n=8) and atopic asthmatics (n=8).

**nonatopic control**

**atopic asthmatic**

**EGFR**


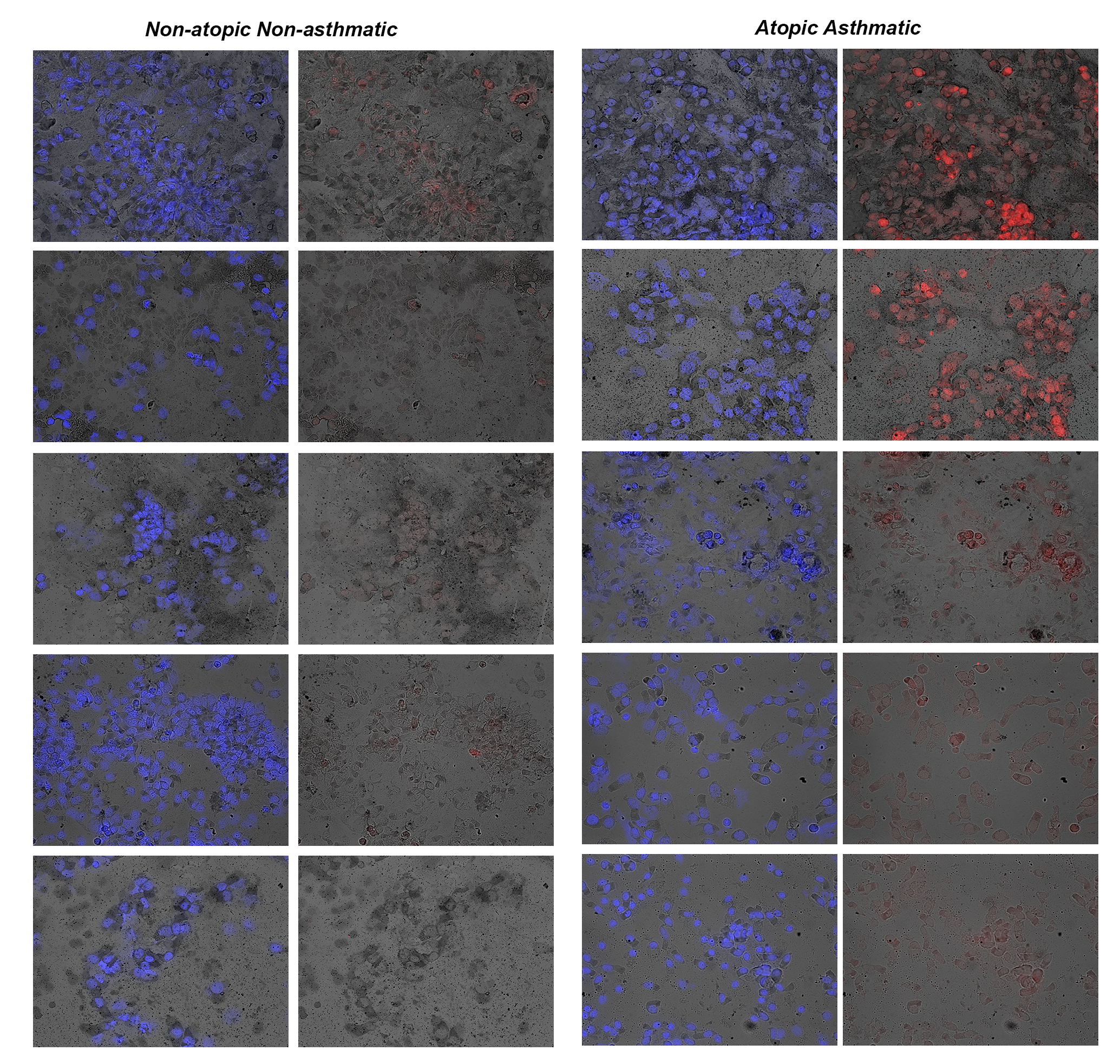


Figure S12. Immunostaining of EGFR (in red) and DAPI (in blue) of bronchial epithelial cells of nonatopic controls (n=5) and atopic asthmatics (n=5).


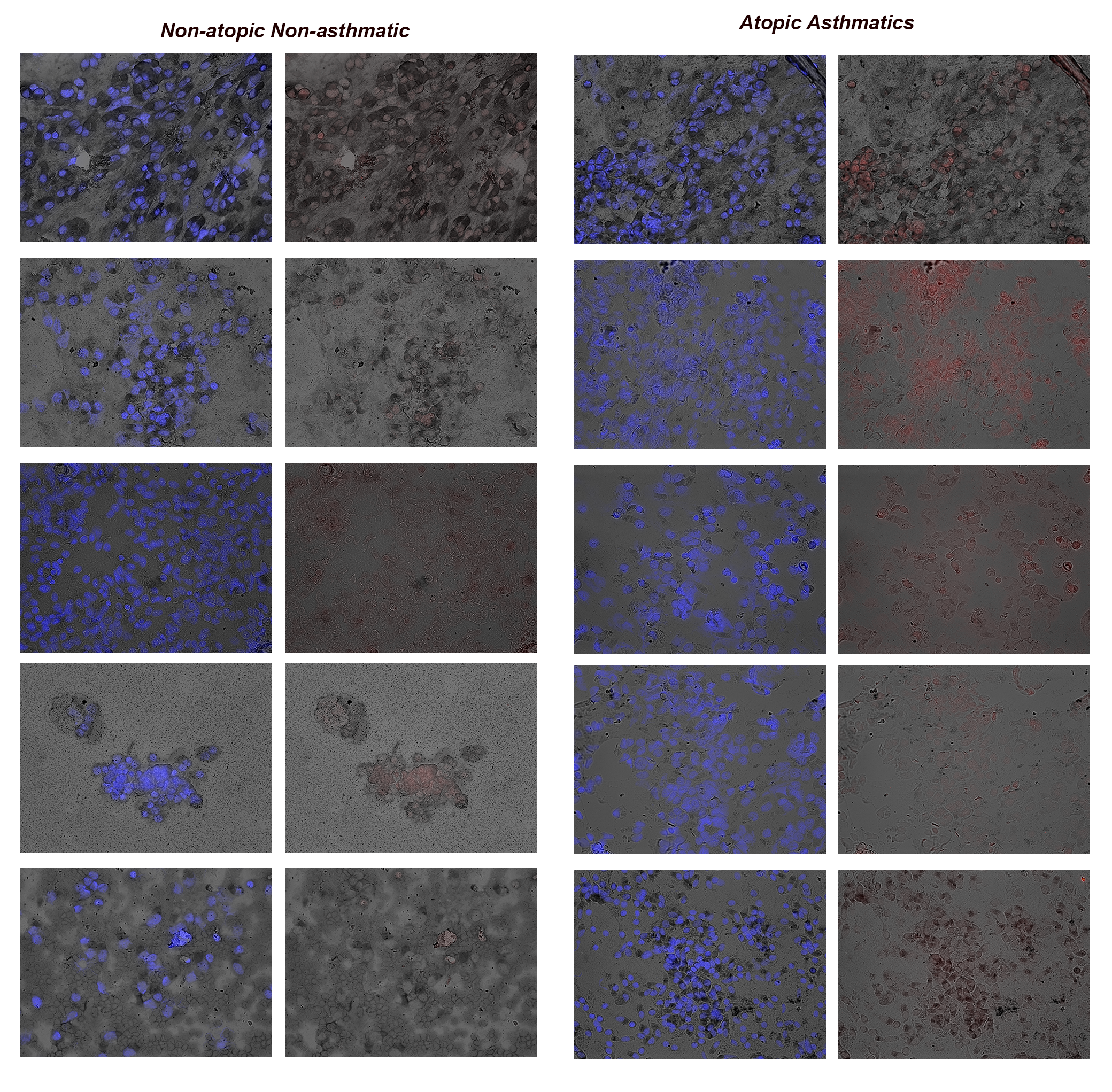


**nonatopic control**

**atopic asthmatic**

**ERBB2**

Figure S13. Immunostaining of ERBB2 (in red) and DAPI (in blue) of bronchial epithelial cells of nonatopic controls (n=5) and atopic asthmatics (n=5).

# References
